# Synthesis of (diarylmethyl)amines using Ni-catalyzed arylation of C(sp^3^)–H bonds†
†Electronic supplementary information (ESI) available: Full experimental and optimization details, characterization of all the synthesized products. See DOI: 10.1039/c5sc01589h



**DOI:** 10.1039/c5sc01589h

**Published:** 2015-06-12

**Authors:** José A. Fernández-Salas, Enrico Marelli, Steven P. Nolan

**Affiliations:** a EaStCHEM School of Chemistry , University of St Andrews , St Andrews , KY16 9ST , UK . Email: stevenpnolan@gmail.com; b Chemistry Department , College of Science , King Saud University , Riyadh 11451 , Saudi Arabia

## Abstract

The first nickel catalyzed deprotonative cross coupling between C(sp^3^)–H bonds and aryl chlorides is reported, allowing the challenging arylation of benzylimines in the absence of directing group or stoichiometric metal activation. This methodology represents a convenient access to the (diarylmethyl)amine moiety, which is widespread in pharmaceutically relevant compounds.

## Abstract 2

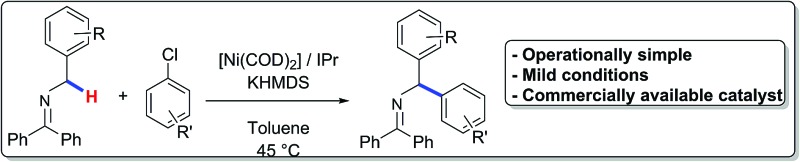
The first nickel catalyzed deprotonative cross coupling between C(sp^3^)–H bonds and aryl chlorides is reported, allowing the challenging arylation of benzylimines in the absence of directing group or stoichiometric metal activation.

## 

Cross-coupling catalysis holds a preferred position in the synthetic chemist's arsenal as it provides a myriad of options for the efficient and user-friendly access to organic motifs that are otherwise difficult or impossible to obtain.^1^ In this context efforts have been devoted to extend the use of cross-coupling to the functionalization of C–H bonds, as this highly attractive strategy leads to an atom-economical formation of new bonds while generating minimal waste.^2^ The use of directing groups and/or activated C–H bonds in this chemistry has been thoroughly studied.^3–5^ In contrast, the use of less acidic C(sp^3^)–H pro-nucleophiles in the absence of any directing group^6,7^ has proven more challenging, and such examples remain scarce.^8^

**Scheme 1:**
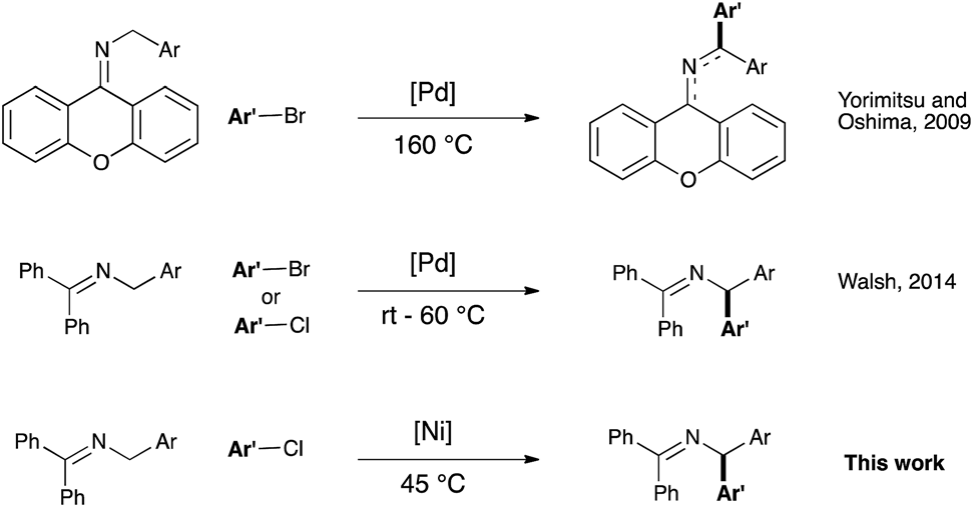
Context of the present work.

The arylation of benzylamines-derived imines belongs to this reaction class. It was initially reported by Yorimitsu and Oshima^9^ in 2008 and more recently alternative protocols have been described.^10^ As most of the deprotonative cross couplings reported to date, these protocols are based on palladium catalysis (see Scheme 1). In recent years, attention has focused on the use of less expensive and more earth-abundant first-row transition metals as catalysts.^11^ Amongst these, nickel has long been used in a number of industrial applications,^12^ and its utility as a powerful catalyst has been *revisited* in several areas of homogenous catalysis, ranging from coupling reactions^13^ to C–H bond functionalization^14^ and small molecules activation.^15^ However, deprotonative functionalization of benzylic C–H bond under nickel catalysis is, to date, unprecedented. As the (diarylmethyl)amine moiety is a well-known pharmacophore motif found in pharmaceuticals,^16^ the development of more sustainable synthetic methodologies towards its synthesis is of significant interest.

We therefore envisioned the use of a Ni–NHC (NHC: *N*-heterocyclic carbene) system, known to be highly active in cross-coupling chemistry,^13d,h,n^ *in lieu* of Pd-based catalysts in this challenging transformation. Our initial hypothesis relied on the existence, for nickel, of a mechanistically closely related process to palladium (see Scheme 2).

**Scheme 2:**
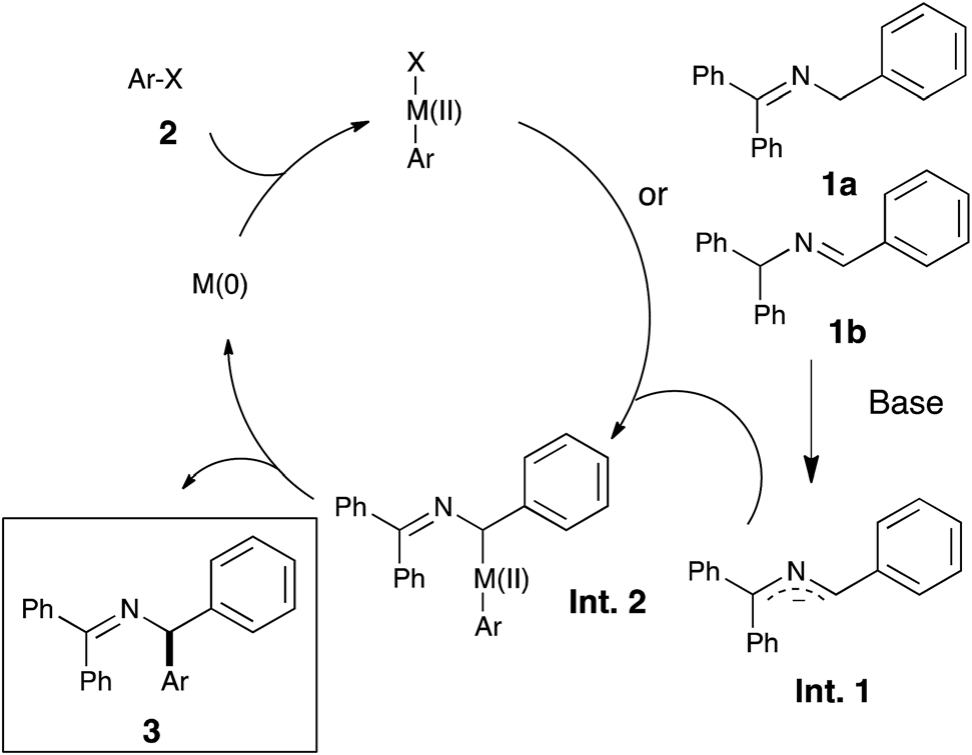
The mechanism of the deprotonative cross coupling of benzylimines.

Our study began with the examination of a model reaction involving **1a** and chlorotoluene (Table 1). The role of the base was examined early on and full conversion and good NMR yields of the product were obtained using a [Ni(COD)_2_](**1**)/IPr catalytic system in toluene (see Fig. 1) when potassium hexamethyldisilylamide (KHMDS) was used as base. All other bases tested gave no conversion to the desired product (for full solvent-base system optimization, see the ESI†).^17^

**Fig. 1:**
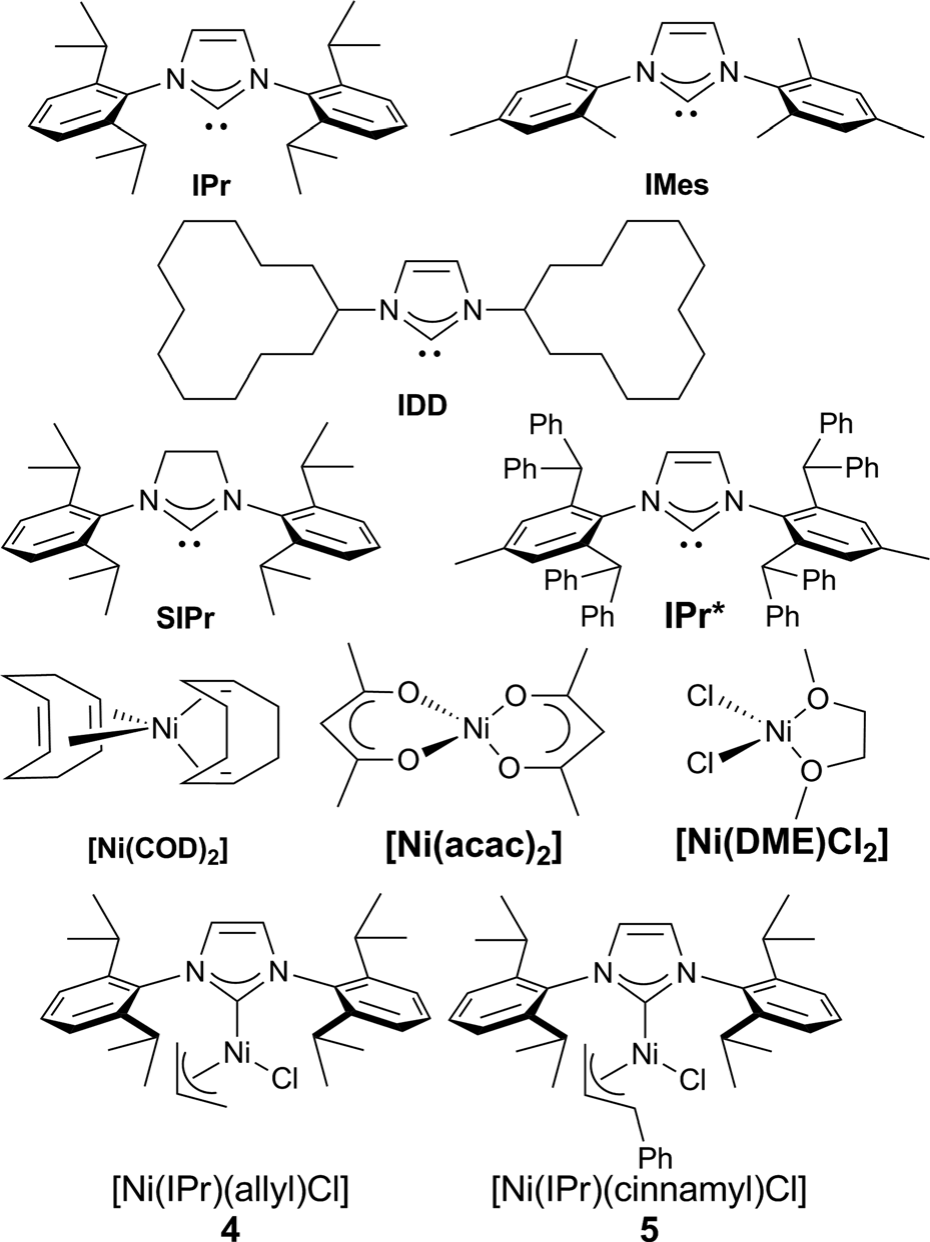
Ligands and complexes tested.

We proceeded to examine the influence of the ligand: the use of smaller NHCs resulted in poor or no conversion (entries 2–3), while SIPr (entry 4) gave only a moderate yield. The use of the very bulky IPr* ligand, which usually provides the best outcome when employed in cross coupling chemistry,^18^ resulted in a lower yield (entry 5). As we identified IPr as the optimal ligand, in our initial reaction, the IPr-bearing well-defined catalysts **4** and **5** (entries 6–7) were tested. In contrast to previous examples of Ni–NHC catalyzed reactions,^13n,15d^ both pre-catalysts gave poorer results compared to the *in situ* prepared [Ni(COD)_2_]/IPr system. We suspect the lower efficiency shown by the preformed pre-catalysts is due to the inability of the 2-azaallyl anion to effectively activate the Ni(ii) center. This is an issue we are currently addressing in the design and synthesis of novel nickel-based pre-catalysts. To complete the optimization, temperature effects were examined and yields decreased with higher temperature (entry 8). The concentration could be increased to 0.17 M (entry 9), but further increase led to dramatic decrease in yield (entry 10). The optimal metal/ligand ratio was found to be 1 : 2 (entry 11). The use of a representative phosphine ligand, PCy_3_, resulted in no conversion. Similar results were obtained when other Ni sources, such as [Ni(acac)_2_] and [Ni(DME)Cl_2_] were employed. Interestingly, further increasing the amount of ligand completely suppressed the reaction. This result suggests that a monoligated Ni species is possibly the catalytically active species, and large excess of ligand moves the equilibrium towards the more stable but inactive bis-ligated species. Gratifyingly, the relatively mild operating temperature does not lead to the formation of isomeric mixtures. It is important to underline that, contrarily to previous reports,^10a,b^ slow addition of the base is unnecessary, thus making our protocol operationally simple.

**Table 1:** Optimization of the reaction conditions^*a*^

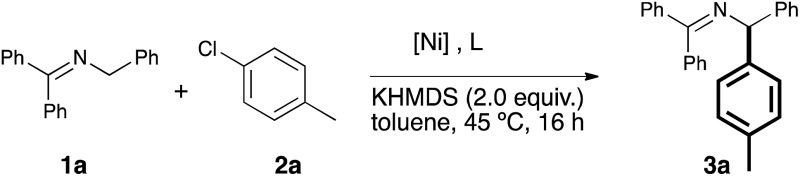				
Entry	[Ni] (5 mol%)	L (mol%)	Conc. (mol L^–1^)	Conv.^*b*^ (yield)^*c*^
1	[Ni(COD)_2_]	IPr (6)	0.10	>95 (81)
2	[Ni(COD)_2_]	IMes (6)	0.10	24
3	[Ni(COD)_2_]	IDD (6)	0.10	—
4	[Ni(COD)_2_]	SIPr (6)	0.10	94 (65)
5	[Ni(COD)_2_]	IPr* (6)	0.10	>95 (60)
6	**4**	—	0.10	70 (45)
7	**5**	—	0.10	>95 (70)
8^*d*^	[Ni(COD)_2_]	IPr (6)	0.10	>95 (72)
9	[Ni(COD)_2_]	IPr (6)	0.17	>95 (85)
10	[Ni(COD)_2_]	IPr (6)	0.25	>95 (53)
11	[Ni(COD)_2_]	IPr (10)	0.17	>95 (93)
12	[Ni(COD)_2_]	PCy_3_ (10)	0.17	—
13	[Ni(acac)_2_]	IPr (10)	0.17	Traces
14	[Ni(DME)Cl_2_]	IPr (10)	0.17	—
15	[Ni(COD)_2_]	IPr (15)	0.17	—

^*a*^Conditions: 4-chlorotoluene (0.25 mmol), imine **1a** (2.0 equiv.), KHMDS (2.0 equiv.), toluene (1.0–2.5 mL), Ni source (2.5–5 mol%), ligand (3–10 mol%), 45 °C, 16 hours.

^*b*^Calculated by G.C. analysis.

^*c*^Yield calculated by NMR analysis using dimethyl malonate as an internal standard.

^*d*^Reaction performed at 60 °C.

Once the optimal reaction conditions were established, we sought to explore the generality of the new protocol by varying the nature of the aryl chloride coupled with **1a** (see Scheme 3). We were pleased to find that both the electron-rich 4-chloroanisole **2b** and the electron-poor chlorides **2c** and **2d** led to high yields of the desired products. The compatibility of functionalized aryl chlorides, bearing functional groups such as amines (**3e**), benzodioxole (**3g**) and relatively sensitive ketone and nitrile derivatives (**3g** and **3h**) was then examined. In all cases, good to very good yields were obtained. The use of heterocyclic (**3i**) and hindered aryl-chlorides (**3j** and **3k**) was also possible; in these cases, complete conversion required a catalyst loading of 7.5 mol%. Compound **3k** was isolated after hydrolysis, as the reaction mixture contained a small impurity that was not possible to remove by column chromatography (see ESI†). The results obtained in the coupling of bulky aryl chlorides clearly improve on previous Pd-based reports. Imines **1b** and **1c**, bearing respectively a 4-methoxyphenyl and a 4-fluorophenyl moiety on the benzylamine starting material afforded the coupling products with chlorobenzene in good yields (see entries **3b-2** and **3c-2**). To highlight some of the limitations of the method, heterocyclic substrates **6–10** proved unsuitable in this transformation.

**Scheme 3:**
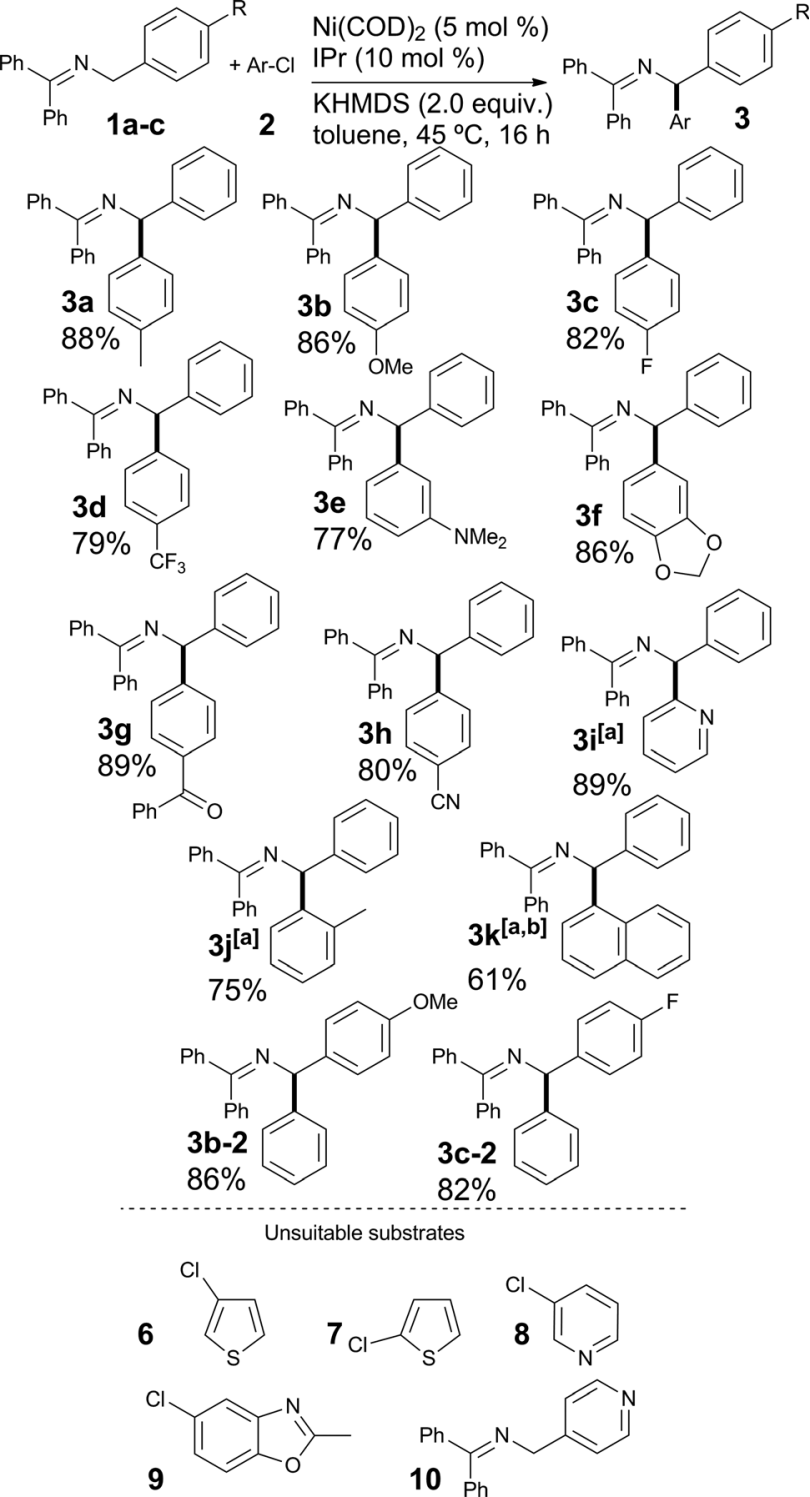
Reaction scope of the Ni-catalyzed arylation of C(sp^3^)–H bonds. Reaction conditions: aryl chloride (**2**) (0.25 mmol), **1** (2.0 equiv.), KHMDS (2.0 equiv.), toluene (1.5 mL), [Ni(COD)_2_] (5 mol%), IPr (10 mol%), 45 °C, 16 h. [a] **4** (7.5 mol%), IPr (15 mol%). [b] Isolated yield of the corresponding ammonium chloride salt.

Encouraged by these results and to further increase the scope and demonstrate the versatility of this catalytic system, the methodology was tested on the commercially available imine **1d**. As the deprotonation of **1a** and **1d** converge to the same intermediate **Int-1**, a unique final product was expected (see Scheme 2). Under the optimized reaction conditions, the desired coupling products were indeed obtained and in very good yields (see Scheme 4).

**Scheme 4:**
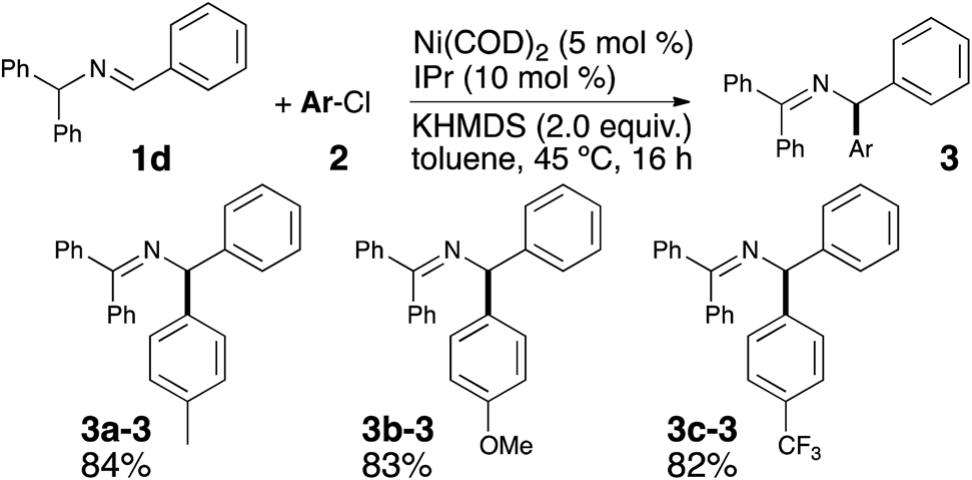
Reaction scope of the Ni-catalyzed arylation of C(sp^3^)–H bonds. Reaction conditions: aryl chloride (**2**) (0.25 mmol), **1** (2.0 equiv.), KHMDS (2.0 equiv.), toluene (1.5 mL), [Ni(COD)_2_] (5 mol%), IPr (10 mol%), 45 °C, 16 h.

In order to shed light on the exact role of the base in this reaction, we performed the alkylation of **1a** using benzyl chloride under the catalytic arylation reaction conditions (toluene, 45 °C) in the absence of the catalyst, using three different bases: KO*t*Bu, NaHMDS and the optimal KHMDS (Table 2). We found that in the presence of a base weaker than the azaallyl anion, such as a *t*-butoxide, lower amounts of alkylated product were observed (entry 3), and the crude NMR analysis showed the formation of significant amounts of side-products, which were absent in the reactions using HMDS containing-bases (entries 1 and 2). This observation led us to test NaHMDS and KHMDS in the absence of any electrophile, finding that while the latter leads only to the formation the expected starting material and the isomerized form **1d** (entry 4), the use of NaHMDS caused side-products to arise (entry 5). No side-products were observed using KHMDS even when the catalytic Ni/IPr system was present in the reaction medium (entry 6). Although further studies are needed to elucidate the mechanism of this reaction, the fact that KHMDS is the only base which cleanly affords the azaallyl indicates that this could be a reasonable explanation for the lack of reactivity of other bases in the catalytic arylation reaction.

**Table 2:** The role of the base^*a*^

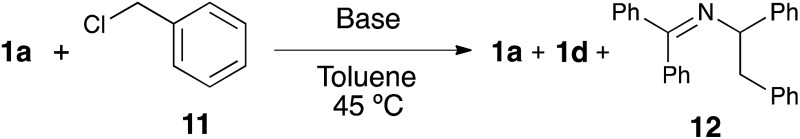				
Entry	Base (equiv.)	**11** (equiv.)	**12** ^*b*^ (%)	Notes
1	KHMDS (2.0)	2.0	81	—
2	NaHMDS (2.0)	0.10	80	—
3	KO*t*Bu (2.0)	0.10	60	Side-products observed
4	KHMDS (0.5)	—	—	Only **1a** and **1d** observed
5	NaHMDS (0.5)	—	24	Side-products observed
6^*c*^	KHMDS (0.5)	—	—	Only **1a** and **1d** observed

^*a*^Conditions: benzyl chloride (0.24 mmol, 1.2 equiv., or none), imine **1a** (0.2 mmol, 1.0 equiv.), base (0.4 mmol or 0.1 mmol, 2.0 equiv. or 0.5 equiv.), toluene (0.6 mmol), 45 °C, 3 hours.

^*b*^Calculated by NMR analysis using dimethyl malonate as an internal standard.

^*c*^Reaction performed in the presence of 5% [Ni(COD)_2_]/10% IPr.

## Conclusions

In summary, we have developed a synthetic methodology to access the (diarylmethyl)amine motif *via* a high yielding Ni-catalyzed coupling between C(sp^3^)–H bonds of benzylimine pro-nucleophiles and aryl chlorides. This work discloses the use of a commercially available Ni-based catalytic system under mild and operationally simple conditions. We hope to soon report on related Ni-catalyzed processes, as well as on the details of the reaction mechanism.
