# Supplementary material for: Synthesis of (diarylmethyl)amines using Ni-catalyzed arylation of C(sp3)–H bonds
Source: Chem Sci. 2015 Jun 12;6(8):4973–7. doi: 10.1039/c5sc01589h (PMC4786957; doi:10.1039/c5sc01589h)
Supplement: Supplementary file 1 [file SC-006-C5SC01589H-s001.pdf]

Electronic Supplementary Material (ESI) for Chemical Science.
This journal is © The Royal Society of Chemistry 2015

Synthesis of (diarylmethyl)amines using Ni-catalyzed arylation of $C(sp^3)$-H bonds

José A. Fernández-Salas, † Enrico Marelli † and Steven P. Nolan* †,‡

Table of Content

I. General considerations 2
II. Synthesis of imines 1a-c. 2
III. Optimization data 2
IV. Experimental procedures 4
V. Characterization data. 5
VI. NMR Spectra 11

I. General considerations

- Aryl chlorides were used as received. Anhydrous, oxygen-free solvents (DMAc, tetrahydrofuran (THF), dimethoxyethane (DME), Dioxane and toluene) and the bases (NaO*t*Bu, KO*t*Bu, KHMDS, LiHMDS, NaHMDS) were stored in a glovebox and used as received. *N*-Benzylidene-*N*-(diphenylmethyl)amine (**1d**) was purchased from Sigma Aldrich and used as received. The well defined Ni complexes [1] and the benzylimine substrates [2] were prepared according to previously reported procedures.
- Flash chromatography was performed on silica gel 60 Å pore diameter and 40-63 µm particle size.
- ^{1}H, ^{13}C and ^{19}F Nuclear Magnetic Resonance (NMR) spectra were recorded on a Bruker- 300, 400 or 500 MHz spectrometer at ambient temperature in CD_3OD or $CDCl_3$. Chemical shifts (δ) are reported in ppm, relative to the solvent residual proton peak CD_3OD (3.31 ppm for ^{1}H and 49.0 ppm for ^{13}C) and $CDCl_3$ (7.26 ppm for ^{1}H and 77.00 ppm for ^{13}C). For ^{19}F NMR, chemical shifts refer to an external calibration using $CFCl_3$ (δ = 0.00 ppm). Data for ^{1}H NMR are reported as follows: chemical shift, multiplicity (s = singlet, d = doublet, t = triplet, br = broad signal, m = multiplet), coupling constants (*J*) in Hz and integration.
- HRMS analysis were performed at the EPSRC UK National Mass Spectrometry Facility (NMSF), Swansea.
- All isolated yields are an average of two runs.

II. Synthesis of imines 1a-c.

Benzophenone (3.6 g, 20 mmol), $NaHCO_3$ (8.4 g, 0.1 mol), the corresponding benzyl amine (21 mmol) and activated molecular sieves (4Å) were weighted in a round bottom flask under dry conditions. Then, dry toluene (40 mL) was added. The reaction was then stirred for 16 hours at 90°C. After this time, the mixture was filtered through celite. The desired imine was obtained pure after recrystallization (AcOEt:*n*-hexane).

III. Optimization data

Table 1: Selection of the precatalyst.

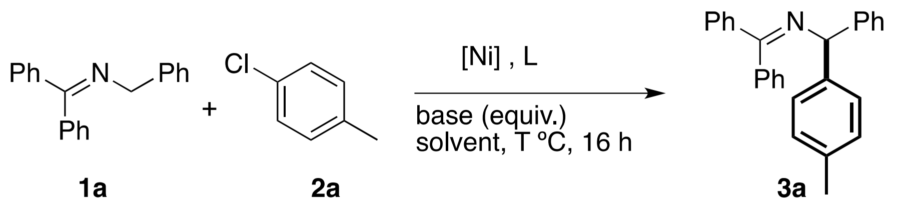

1a **2a** **3a**

Entry	[Ni] (mol %)/NHC	Base	Solvent (mL)	T° C	Conv% (NMR

[1] (1) Dible, B. R.; Sigman, M. S. *J. Am. Chem. Soc.* **2003**, *125*, 872–873.

	(mol %)	(equiv.)			yield)[a]
1	[Ni(COD)$_2$] (5)/IPr (6)	NaO*t*Bu (2.0)	Toluene (2.5)	45	-
2	[Ni(COD)$_2$] (5)/IPr (6)	KO*t*Bu (2.0)	Toluene (2.5)	45	-
3	[Ni(COD)$_2$] (5)/IPr (6)	NaHMDS (2.0)	Toluene (2.5)	45	-
4	[Ni(COD)$_2$] (5)/IPr (6)	LiHMDS (2.0)	Toluene (2.5)	45	-
6	[Ni(COD)$_2$] (5)/IPr (6)	KHMDS (1.5)	Toluene (2.5)	45	>95 (75)
7	[Ni(COD)$_2$] (5)/IPr (6)	KHMDS (2.5)	Toluene (2.5)	45	>95 (72)
8	[Ni(COD)$_2$] (5)/IPr (6)	KHMDS (2.0)	THF (2.5)	45	>95 (49)
9	[Ni(COD)$_2$] (5)/IPr (6)	KHMDS (2.0)	DME (2.5)	45	27
10	[Ni(COD)$_2$] (5)/IPr (6)	KHMDS (2.0)	DMAc (2.5)	45	-
11	[Ni(COD)$_2$] (5)/IPr (6)	KHMDS (2.0)	Dioxane (2.5)	45	63
12	[Ni(COD)$_2$] (5)/IPr (6)	KHMDS (2.0)	Toluene (2.5)	45	>95 (81)
13	[Ni(COD)$_2$] (5)/SIPr (6)	KHMDS (2.0)	Toluene (2.5)	45	94 (65)
14	[Ni(COD)$_2$] (5)/IPr* (6)	KHMDS (2.0)	Toluene (2.5)	45	>95 (60)
15	[Ni(COD)$_2$] (5)/IDD (6)	KHMDS (2.0)	Toluene (2.5)	45	-
16	[Ni(COD)$_2$] (5)/IMes (6)	KHMDS (2.0)	Toluene (2.5)	45	24
17	[Ni(IPr)(cin)Cl] (5)	KHMDS (2.0)	Toluene (2.5)	45	>95 (70)
18	[Ni(IPr)(all)Cl] (5)	KHMDS	Toluene (2.5)	45	70 (45)

		(2.0)			
19	[Ni(COD)$_2$] (5)/IPr (6)	KHMDS (2.0)	Toluene (2.5)	60	>95 (72)
20	[Ni(COD)$_2$] (5)/IPr (6)	KHMDS (2.0)	Toluene (1)	45	>95 (53)
21	[Ni(COD)$_2$] (5)/IPr (6)	KHMDS (2.0)	Toluene (1.5)	45	>95 (85)
22	[Ni(COD)$_2$] (2.5)/IPr (3)	KHMDS (2.0)	Toluene (1.5)	45	>95 (72)
**23**	**[Ni(COD)$_2$] (5)/IPr (10)**	**KHMDS (2.0)**	**Toluene (1.5)**	**45**	**>95 (93)**
24	[Ni(COD)$_2$] (5)/IPr (7.5)	KHMDS (2.0)	Toluene (1.5)	45	>95 (89)
25	[Ni(acac)$_2$] (5)/IPr (10)	KHMDS (2.0)	Toluene (1.5)	45	traces
26	[Ni(DME)Cl$_2$] (5)/IPr (10)	KHMDS (2.0)	Toluene (1.5)	45	-
27	[Ni(IPr)(cin)Cl] (5)/ IPr (5)	KHMDS (2.0)	Toluene (2.5)	45	>95 (70)

Reaction conditions: **1a** (0.5 mmol, 2.0 equiv), 4-chlorotoluene (0.25 mmol, 1 equiv). [a] Conversion calculated by NMR. NMR yield obtained using dimethylmalonate as internal reference.

IV. Experimental procedures

General procedure for the arylation of imines. Inside a glovebox, [Ni(COD)$_2$] (0.0125 mmol,), IPr (10 or 15 mg, 2 equiv. with respect of Ni) the selected imine (0.5 mmol, 2 equiv.), KHMDS (100 mg, 0.5 mmol) were weighted in a screw cap vial equipped with a stirring bar. The aryl chloride (0.25 mmol, 1 equiv.) was added at this stage, if solid. The vial was sealed and carried out from the glovebox, where and the aryl chloride, if liquid, and the dry, degassed solvent (toluene, 1.5 ml) were added. The reaction was then stirred for 16 hours at 45°C. The reaction was then quenched adding some drops of water and filtered through Mg_2SO_4. After checking the NMR, the crude was absorbed on silica previously basified (stirring it overnight with 2% triethylamine in pentane) and purified by flash chromatography (typically with a pentane / diethyl ether = 95 / 5 eluent mixture) to afford the desired product.

Procedure for the hydrolysis of 3k. HCl 1N in diethyl ether (1 mL) was added to the solution of imine **3k** (39.7 mg, 0.1 mmol) in THF (1 mL) at 0°C. The solution was warmed to room temperature, stirred at room temperature and monitored by TLC until all the imine was consumed. The THF was evaporated under vacuum. Another 1 mL HCl (1N) was added and a white precipitate was observed. The white solid was filtered and washed with cold Et_2O (1.0 mL×3). After drying under vacuum for 12 h, the hydrochloride salt was obtained as a white solid (25.4 mg, 99% yield).

Procedure for the benzylation experiments of 1a (table2, entries 1-3). Inside a glovebox, **1a** (54 mg, 0.2 mmol, 1.0 equiv.) and the base (2.0 equiv.) were weighted in a screw cap vial equipped with a stirring bar. Outside the glovebox, toluene (0.6 mL) and benzyl chloride (0.25 µL, 2.4 mmol, 1.2 equiv.) were added, and the reaction was then stirred at 45 °C for 3 hours. The reaction was then quenched with 2 drops of water, filtered thru $MgSO_4$ and dried under vacuum. The yield was then assessed *via* quantitative ^{1}H-NMR, using diethylmalonate as internal standard.

Procedure for the bases test (table 2, entries 4-6). Inside a glovebox, **1a** (54 mg, 0.2 mmol, 1.0 equiv.) and the base (0.5 equiv.) were weighted in a screw cap vial equipped with a stirring bar. Outside the glovebox, dry degassed toluene (0.6 mL) was added using a syringe thru the septum, and the reaction was then stirred at 45 °C for 3 hours. The reaction was then quenched with 2 drops of water, filtered thru $MgSO_4$, dried under vacuum and analyzed via ^{1}H-NMR.

V. Characterization data.

N-(Diphenylmethylene)-1-phenyl-1-(*p*-tolyl)methanamine. (3a)[2]

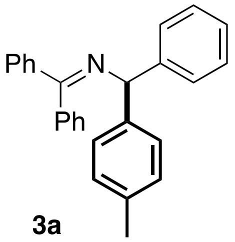

3a

Chemical Formula: $C_{27}H_{23}N$
Molecular Weight: 361,48

From **1a** and 4-chlorotoluene, 5% [Ni] loading, isolated yield: 318 mg, 88%.

From **1d** and 4-chlorotoluene, 5% [Ni] loading, isolated yield: 304 mg, 84%.

^{1}H NMR (400 MHz, $CDCl_3$): δ 7.51 (m, 2H), 7.45-7.41 (m, 3H), 7.38-7.27 (m, 7H), 7.22-7.18 (m, 3H), 7.10-7.07 (m, 4H), 5.52 (s, 2H), 2.31 (s, 3H).

^{13}C NMR (100 MHz, $CDCl_3$): δ 166.6, 141.9, 139.9, 136.7, 136.1, 130.0, 129.0, 128.7, 128.4, 128.4, 128.3, 127.9, 127.8, 127.5, 127.4, 126.6, 69.6, 21.1.

---

[2] Li, M.; Yucel, B.; Adrio, J.; Bellomo, A.; Walsh, P. J. *Chem. Sci.* **2014**, *5*, 2383–2391.

N-(Diphenylmethylene)-1-(4-methoxyphenyl)-1-phenylmethanamine. (3b)[2]

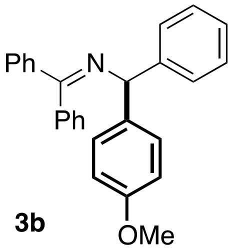

3b

Chemical Formula: $C_{27}H_{23}NO$
Molecular Weight: 377,48

From **1a** and 4-chloroanisole, 5% Ni loading, isolated yield: 325 mg, 86%.

From **1b** and chlorobenzene, 5% Ni loading, isolated yield: 325 mg, 86%.

From **1d** and 4-chloroanisole, 5% Ni loading, isolated yield: 313 mg, 83%.

^{1}H NMR (400 MHz, $CDCl_3$): δ 7.76 (d, J= 7.2 Hz, 2H), 7.45-7.25 (m, 10H), 7.22-7.18 (m, 3H), 7.11-7.08 (m, 2H), 7.10-7.07 (m, 4H), 6.83 (d, *J*=8.8 Hz, 2H), 5.53 (s, 1H), 3.78 (s, 3H).

^{13}C NMR (100 MHz, $CDCl_3$): δ 166.57, 158.31, 145.11, 139.85, 137.15, 136.73, 129.98, 128.71, 128.58, 128.42, 128.37, 128.27, 127.96, 127.73, 127.44, 126.56, 113.70, 69.19, 55.20.

N-(Diphenylmethylene)-1-(4-fluorophenyl)-1-phenylmethanamine. (3c)[2]

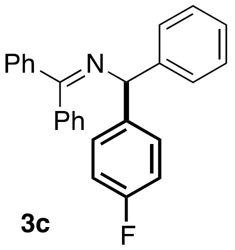

3c

Chemical Formula: $C_{26}H_{20}FN$
Molecular Weight: 365,44

From **1a** and 4-fluorochlorobenzene, 5% [Ni] loading, Isolated yield : 299 mg, 82%.

From **1c** and chlorobenzene, 5% [Ni] loading, Isolated yield : 298 mg, 82%.

From **1d** and 4-fluorochlorobenzene, 5% [Ni] loading, Isolated yield : 298 mg, 82%.

^{1}H NMR (400 MHz, $CDCl_3$): δ 7.77 (m, 2H), 7.47-7.44 (m, 3H), 7.40-7.30 (m, 2H), 7.30-7.26 (m, 6H), 7.24-7.18 (m, 1H), 7.10-7.06 (m, 2H), 7.00-6.94 (m, 2H), 5.55 (s, 1H).

^{13}C NMR (100 MHz, $CDCl_3$): δ 167.1, 162.0 (d, $^1J_{C-F}$= 243.0 Hz), 144.7, 140.6 (d, $^4J_{C-F}$= 3.0 Hz), 139.7, 136.6, 130.1, 129.0 (d, $^3J_{C-F}$= 7.8 Hz), 128.7, 128.5, 128.4, 128.4, 128.0, 127.6, 127.4, 126.8, 115.0 (d, $^2J_{C-F}$= 31.2 Hz), 69.3.

^{19}F NMR (376.8 MHz, $CDCl_3$): δ -116.4

N-(Diphenylmethylene)-1-phenyl-1-(4-(trifluoromethyl)phenyl)methanamine. (3d)[2]

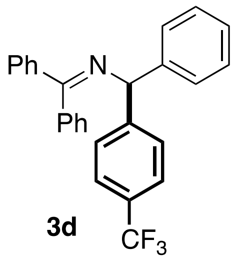

3d

Chemical Formula: $C_{27}H_{20}F_3N$
Molecular Weight: 415,45

5% [Ni] loading. Isolated yield: 328 mg, 79%.

^{1}H NMR (400 MHz, $CDCl_3$) : δ 7.77 (m, 2H), 7.55 (app. d, J= 8.4 Hz, 2H), 7.48-7.26 (m, 12H), 7.25-7.21 (m, 1H), 7.24-7.18 (m, 1H), 7.09-7.05 (m, 2H), 5.61 (s, 1H).

^{13}C NMR (100 MHz, $CDCl_3$) : δ 167.7, 148.8, 144.0, 139.5, 136.5, 130.3, 129.0, 128.8, 128.7, 128.5, 128.0, 127.8, 127.6, 127.0, 125.3 (q, $J_{C\text{-}F}$= 3.4 Hz), 123.4 (q, $J_{C\text{-}F}$= 270.1 Hz), 69.5

^{19}F NMR (376.8 MHz, $CDCl_3$) : δ -62.4

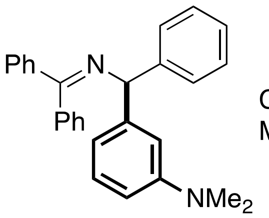

3-(((Diphenylmethylene)amino)(phenyl)methyl)-*N*,*N*-dimethylaniline. (3e)[3] 3e

5% [Ni] loading. Isolated yield: 301 mg, 77%.

^{1}H NMR (400 MHz, $CDCl_3$) : δ 7.75 (m, 2H), 7.45-7.42 (m, 3H), 7.37-7.33 (m, 5H), 7.29-7.25 (m, 3H), 7.20-7.08 (m, 4H), 6.74 (m, 1H), 6.70 (m, 1H), 6.59 (ddd, *J*= 8.4 Hz, 2.8 Hz, 0.8 Hz, 1H),5.51 (s, 2H), 2.90 (s, 6H).

^{13}C NMR (125 MHz, $CDCl_3$) : δ 166.52, 150.59, 145.55, 145.03, 139.95, 136.75, 129.89, 128.92, 128.72, 128.36, 128.28, 128.19, 127.92, 127.87, 127.48, 126.47, 116.20, 112.01, 111.04, 77.00, 70.21, 40.65.

1-(Benzo[*d*][1,3]dioxol-5-yl)-*N*-(diphenylmethylene)-1-phenylmethanamine. (3f)

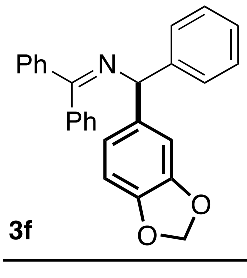

3f

Chemical Formula: $C_{27}H_{21}NO_2$
Molecular Weight: 391,46

[3] Li, M.; Berritt, S.; Walsh, P. J. *Org. Lett.* **2014**, *16*, 4312–4315.

5% [Ni] loading. Isolated yield: 337 mg, 86%.

HRMS: [M-H]$^+$, m/z calculated : 390.1489; observed: 390.1481.

^{1}H NMR (400 MHz, $CDCl_3$): δ 7.79 (m, 2H), 7.48-7.28 (m, 11H), 7.26-7.20 (m, 1H), 7.13 (d, *J*= 2 Hz, 1H), 7.11 (m, 1H), 6.95 (d, *J*= 1,6 Hz, 1H), 6.78 (dd, J^1=8.4 Hz, J^2=1.6 Hz, 1H), 6.74 (d, *J*= 8 Hz, 1H), 5.92 (s, 2H), 5.51 (s, 1H).

^{13}C NMR (100 MHz, $CDCl_3$): δ 166.75, 147.58, 146.23, 144.91, 139.74, 138.96, 136.62, 130.05, 128.71, 128.46, 128.38, 128.30, 127.97, 127.68, 127.33, 126.66, 120.43, 108.26, 107.91, 100.79, 69.44.

4-(((Diphenylmethylene)amino)(phenyl)methyl)phenyl)(phenyl)methanone. (3g)

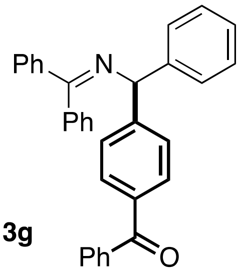

3g

Chemical Formula: $C_{33}H_{25}NO$
Molecular Weight: 451,56

5% [Ni] loading. Isolated yield: 402 mg, 89%.

HRMS: [M-H]$^+$, m/z calculated : 450.1852; observed: 450.1548

^{1}H NMR (500 MHz, $CDCl_3$): δ 7.85-7.80 (m, 6H), 7.59 (t, *J*= 7.5 Hz, 1H), 7.53-7.47 (m, 7H), 7.45-7.39 (m, 5H), 7.35 (t, *J*= 7.5 Hz, 2H), 7.29-7.24 (m, 1H), 7.14-7.11 (m, 2H), 5.69 (s, 2H), 2.31 (s, 3H).

^{13}C NMR (125 MHz, $CDCl_3$): δ 196.40, 167.60, 149.59, 144.09, 139.53, 137.67, 132.22, 130.32, 130.25, 129.96, 128.73, 128.62, 128.47, 128.16, 128.03, 127.59, 127.52, 127.36, 126.97, 69.67.

4-(((Diphenylmethylene)amino)(phenyl)methyl)benzonitrile. (3h)[2]

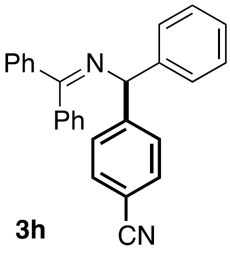

3h

Chemical Formula: $C_{27}H_{20}N_2$
Molecular Weight: 372,46

5% [Ni] loading. Isolated yield: 297 mg, 80%.

^{1}H NMR (400 MHz, $CDCl_3$): δ 7.76-7.73 (m, 2H), 7.57 (d, J= 8.4 Hz, 2H), 7.47-7.36 (m, 15H), 7.06-7.3 (m, 2H), 5.57 (s, 2H).

^{13}**C NMR** (100 MHz, $CDCl_3$): δ 168.12, 150.21, 143.57, 139.34, 136.31, 132.22, 130.44, 128.74, 128.61, 128.56, 128.19, 128.10, 127.50, 127.48, 127.23, 119.01, 110.46, 69.47.

N-(Diphenylmethylene)-1-phenyl-1-(pyridin-2-yl). (3i)

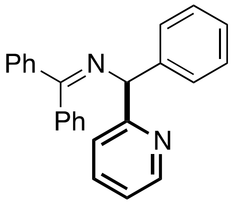

Chemical Formula: $C_{25}H_{20}N_2$
Molecular Weight: 348,44

3i

7.5% [Ni] loading. Isolated yield: 310 mg, 89%.

HRMS: $[M\text{-}H]^+$, m/z calculated : 347.1543; observed: 347.1539.

^{1}**H NMR** (500 MHz, $CDCl_3$): δ 8.47 (d, J= 4.5 Hz, 1H), 7.83-7.77 (m, 3H), 7.67 (dt, *J*= 7.5, 1.5 Hz, 1H), 7.42-7.35 (m, 8H), 7.28 (t, *J*= 7.5 Hz, 2H), 7.22-7.18 (m, 1H), 7.12-7.09 (ddd, *J*= 7.5 Hz, 4 Hz, 1 Hz, 1H), 7.07-7.04 (m, 2H), 5.77 (s, 2H).

^{13}**C NMR** (125 MHz, $CDCl_3$) : δ 167.93, 163.87, 148.88, 143.96, 139.82, 136.66, 136.33, 130.15, 128.77, 128.58, 128.42, 128.36, 128.00, 127.72, 127.46, 126.82, 122.03, 121.80, 71.89.

N-(Diphenylmethylene)-1-phenyl-1-(*o*-tolyl)methanamine. (3j)[2]

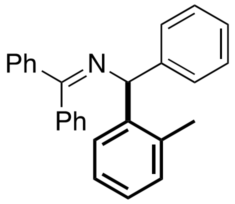

Chemical Formula: $C_{27}H_{23}N$
Molecular Weight: 361,48

3j

^{1}**H NMR** (400 MHz, $CDCl_3$): δ 7.77-7.74 (m, 2H), 7.71 (d, J= 7.6 Hz, 1H), 7.46-7.18 (m, 13H), 7.09-7.06 (m, 3H), 5.76 (s, 1H), 1.97 (s, 3H).

^{13}**C NMR** (100 MHz, $CDCl_3$) : δ 166.76, 144.00, 142.74, 139.77, 137.01, 135.34, 130.34, 129.99, 128.72, 128.53, 128.43, 128.21, 127.96, 127.62, 126.59, 126.46, 126.06, 66.68, 19.53.

Naphthalen-1-yl(phenyl)methanaminium chloride. (3k)[2]

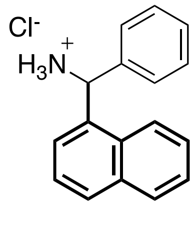

Chemical Formula: $C_{17}H_{16}N^+$
Molecular Weight: 234,32

3k

7.5% [Ni] loading. Isolated yield: 143 mg, 61% (overall yield after hydrolysis)

^{1}H NMR (400 MHz, $CDCl_3$): δ 8.01-7.94 (m, 3H), 7.72-7.63 (m, 2H), 7.56-7.37 (m, 7H), 6.44 (s, 1H).

^{13}C NMR (100 MHz, $CDCl_3$): δ 138.77, 135.55, 131.43, 130.64, 130.30, 130.24, 130.11, 129.13, 128.51, 128.03, 127.40, 126.15, 124.40, 124.15, 56.05.

N-(Diphenylmethylene)-1-phenylmethanamine. (1a)[2]

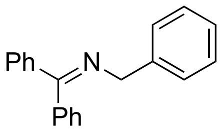

Chemical Formula: $C_{20}H_{17}N$
Molecular Weight: 271,36

^{1}H NMR (400 MHz, $CDCl_3$): δ 7.71-7.65 (m, 2H), 7.51-7.34 (m, 10H), 7.25-7.20 (m, 3H), 4.62 (m, 3H), 7.10-7.07 (m, 4H), 5.52 (s, 2H), 2.31 (s, 3H).

N-(Diphenylmethylene)-1-(4-methoxyphenyl)methanamine. (1b)[2]

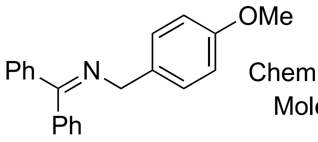

Chemical Formula: $C_{21}H_{19}NO$
Molecular Weight: 301,38

^{1}H NMR (400 MHz, $CDCl_3$): δ 7.69-7.65 (m, 2H), 7.51-7.34 (m, 6H), 7.26-7.19 (m, 4H), 6.89 (m, 2H), 4.55 (s, 2H), 5.52 (s, 2H), 3.80 (s, 3H).

N-(Diphenylmethylene)-1-(4-fluorphenyl)methanamine. (1c)[2]

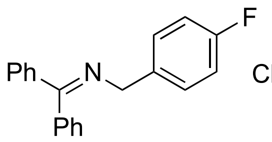

Chemical Formula: $C_{20}H_{16}FN$
Molecular Weight: 289,35

^{1}H NMR (400 MHz, $CDCl_3$): δ 7.72-7.68 (m, 2H), 7.51-7.28 (m, 8H), 7.23-7.19 (m, 2H), 7.05-6.99 (m, 2H), 4.58 (s, 2H).

VI. NMR Spectra

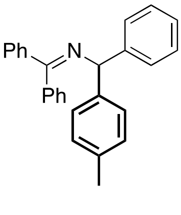

3a

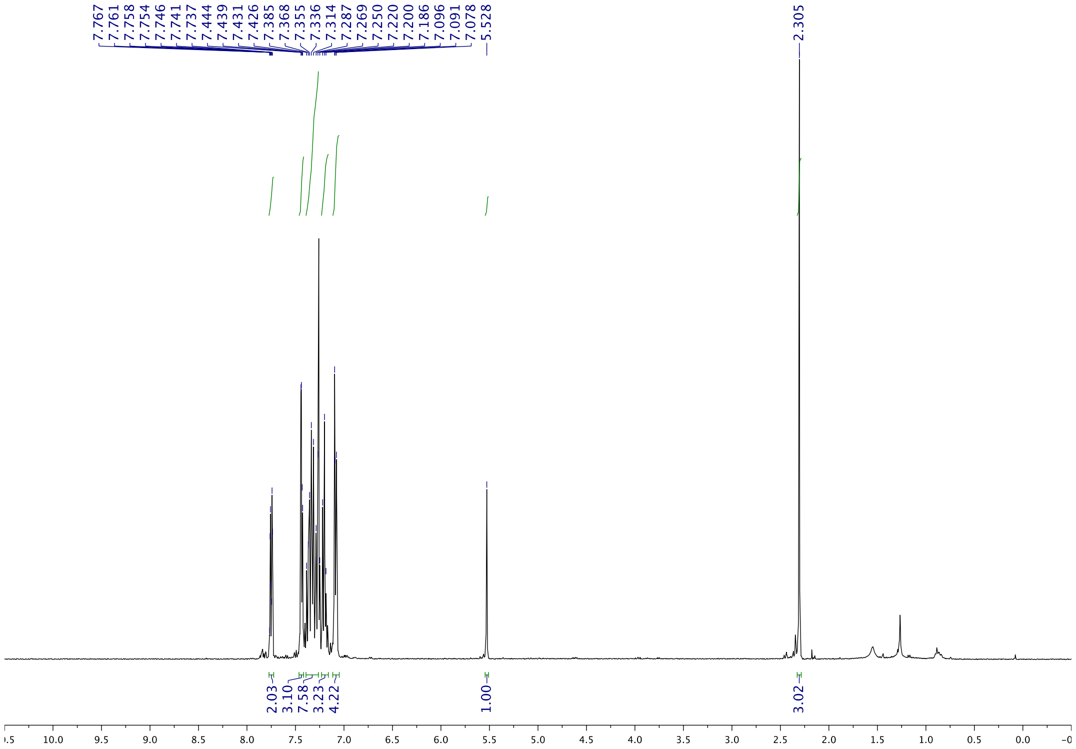

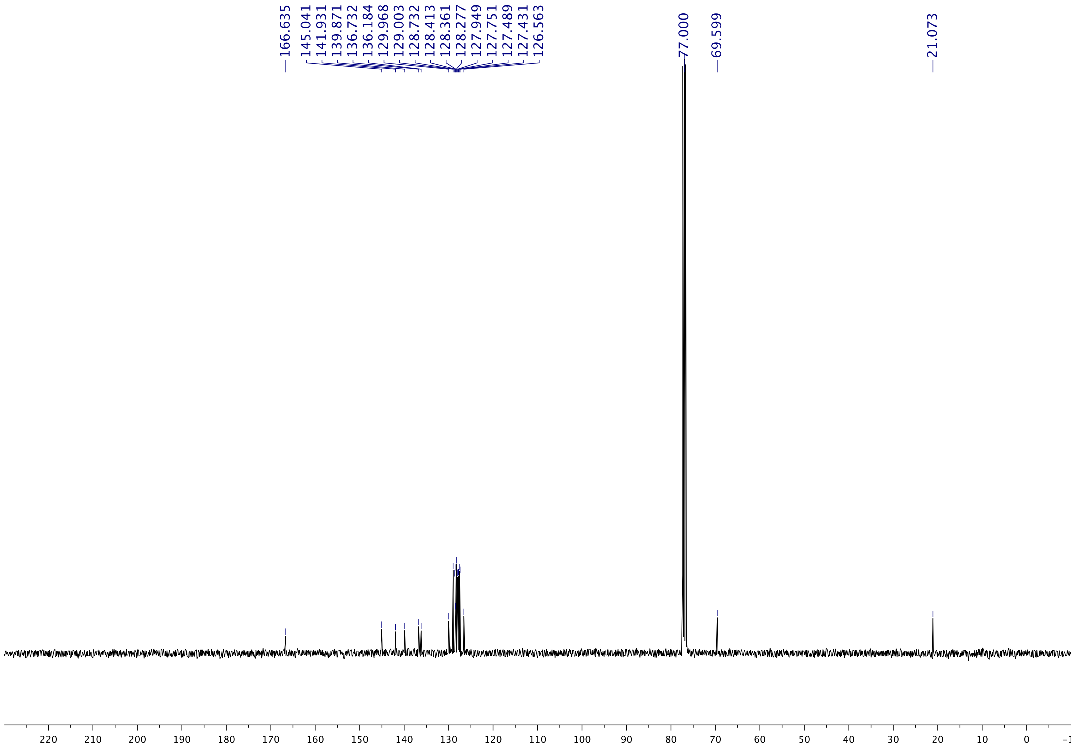

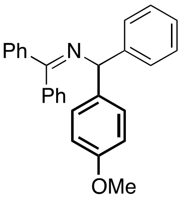

3b

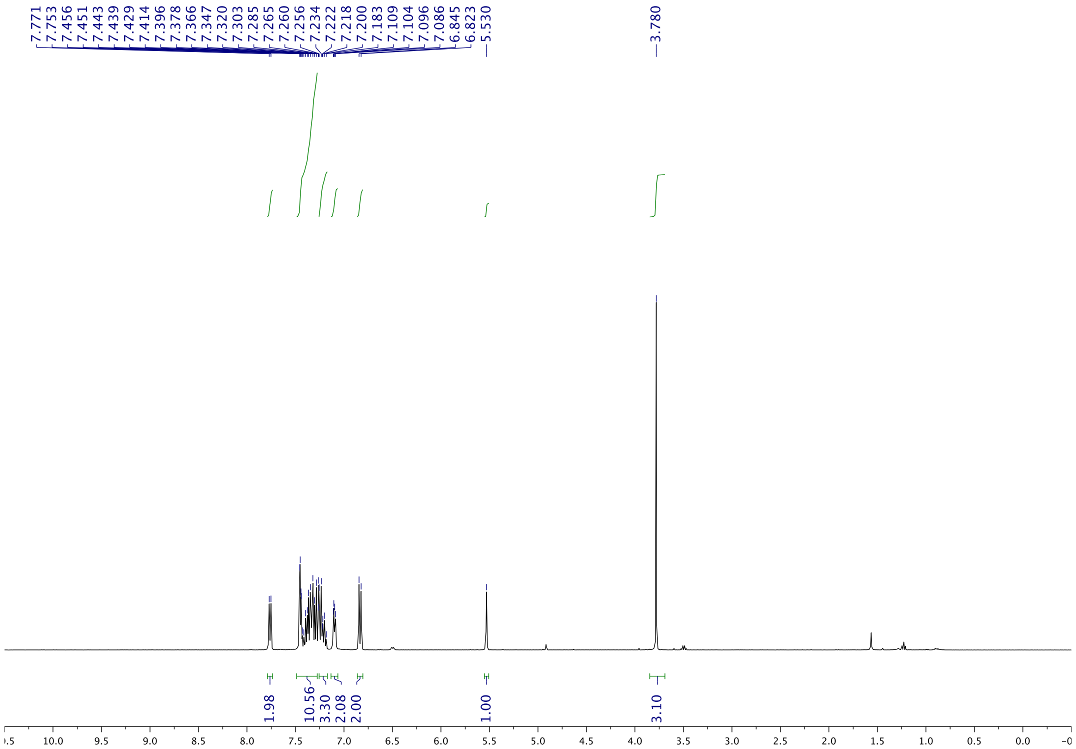

7.771
7.753
7.456
7.451
7.443
7.439
7.429
7.414
7.396
7.378
7.366
7.347
7.320
7.303
7.285
7.265
7.260
7.256
7.234
7.222
7.218
7.200
7.183
7.109
7.104
7.096
7.086
6.845
6.823
5.530
3.780
1.98
10.56
3.30
2.08
2.00
1.00
3.10
10.0
9.5
9.0
8.5
8.0
7.5
7.0
6.5
6.0
5.5
5.0
4.5
4.0
3.5
3.0
2.5
2.0
1.5
1.0
0.5
0.0

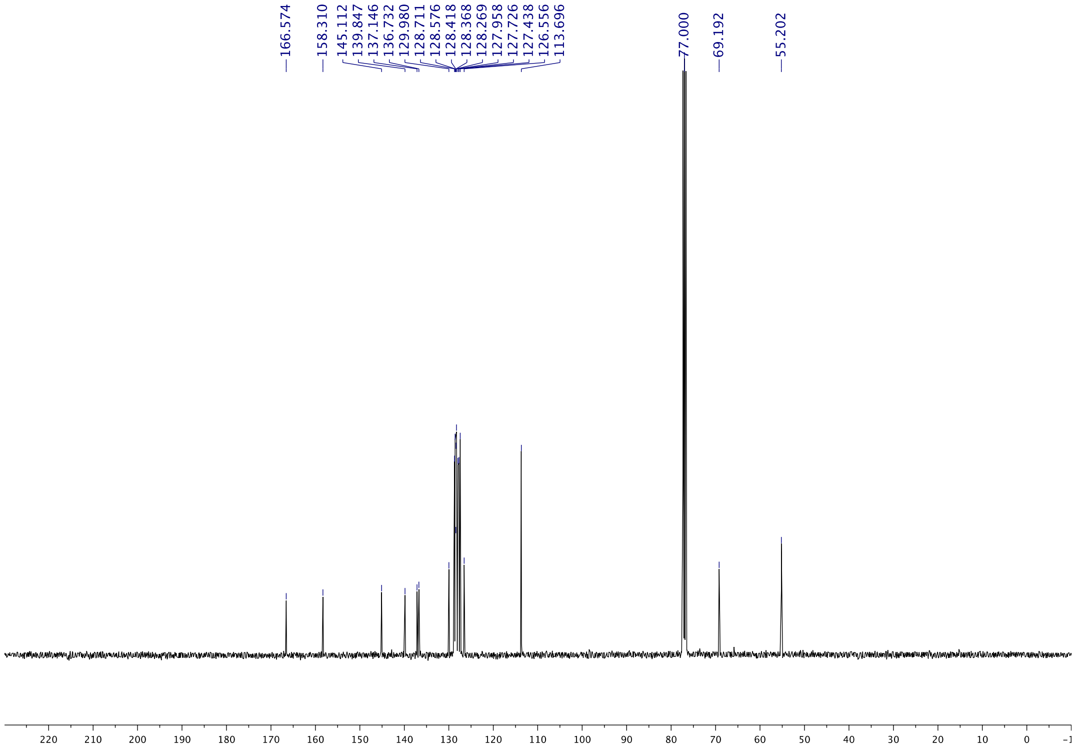

166.574
158.310
145.112
139.847
137.146
136.732
129.980
128.711
128.576
128.418
128.368
128.269
127.958
127.726
127.438
126.556
113.696
77.000
69.192
55.202
220
210
200
190
180
170
160
150
140
130
120
110
100
90
80
70
60
50
40
30
20
10
0

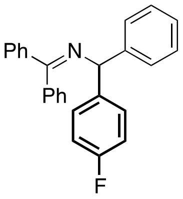
Ph
N
Ph
F

3c

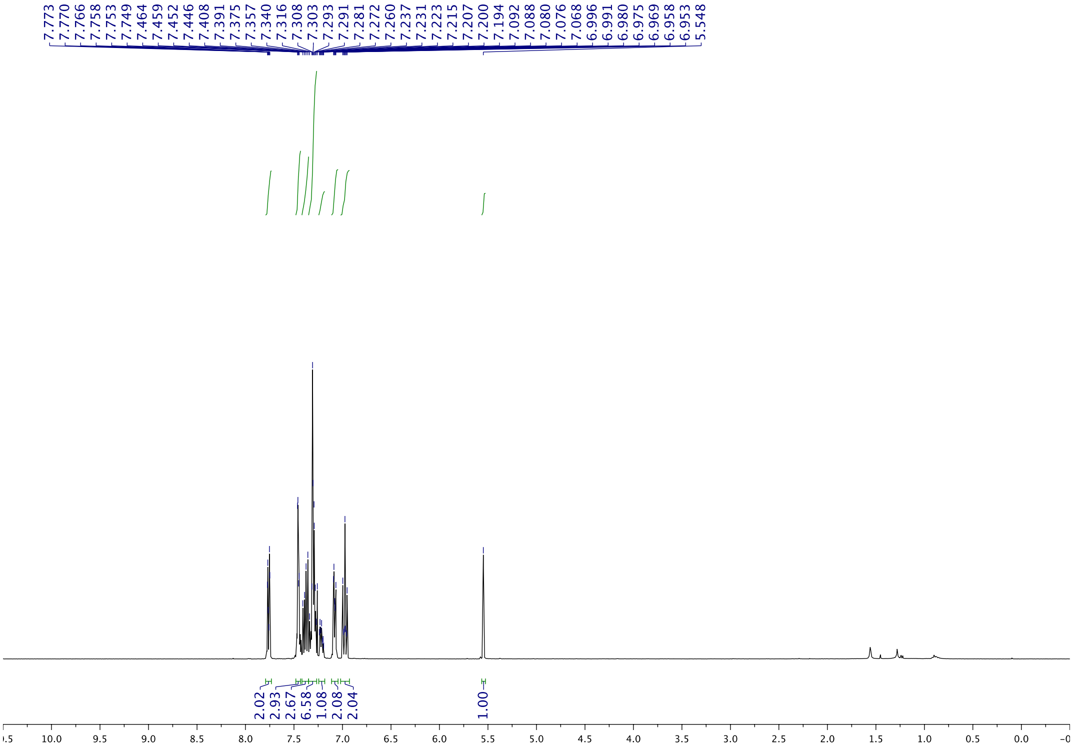
7.773 7.770 7.766 7.758 7.753 7.749 7.464 7.459 7.452 7.446 7.408 7.391 7.375 7.357 7.340 7.316 7.308 7.303 7.293 7.291 7.281 7.272 7.260 7.237 7.231 7.223 7.215 7.207 7.200 7.194 7.092 7.088 7.080 7.076 7.068 6.996 6.991 6.980 6.975 6.969 6.958 6.953 5.548
2.02 2.93 2.67 6.58 1.08 2.08 2.04
1.00
10.0 9.5 9.0 8.5 8.0 7.5 7.0 6.5 6.0 5.5 5.0 4.5 4.0 3.5 3.0 2.5 2.0 1.5 1.0 0.5 0.0

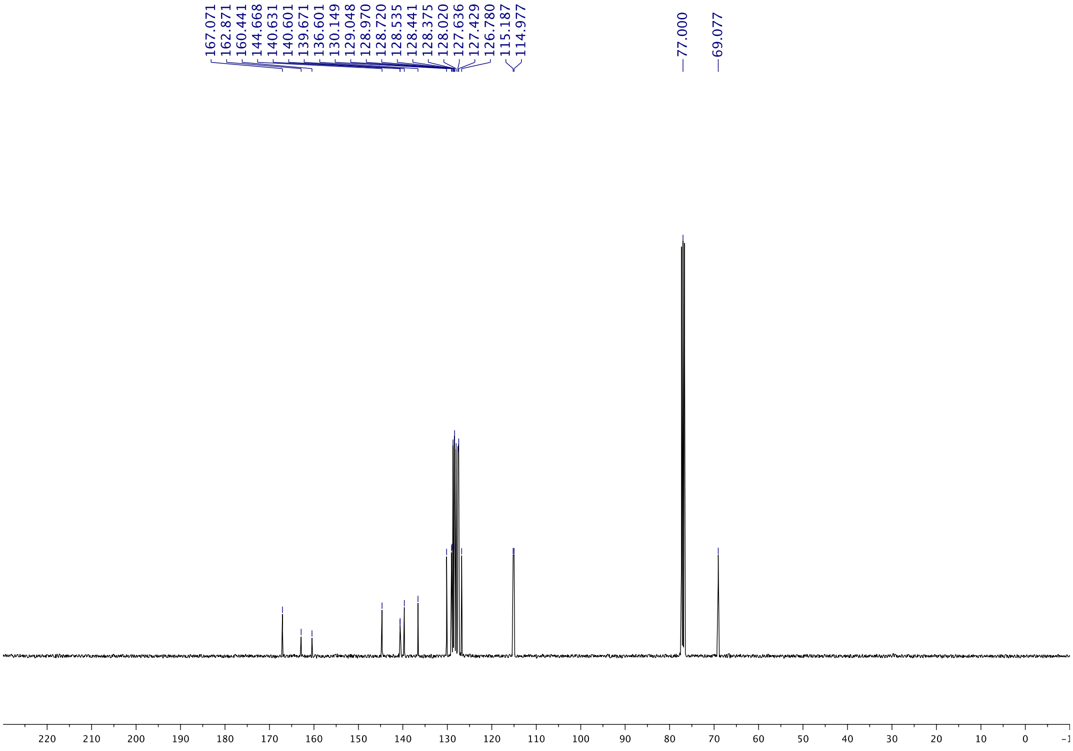
167.071 162.871 160.441 144.668 140.631 140.601 139.671 136.601 130.149 129.048 128.970 128.720 128.535 128.441 128.375 128.020 127.636 127.429 126.780 115.187 114.977
77.000
69.077
220 210 200 190 180 170 160 150 140 130 120 110 100 90 80 70 60 50 40 30 20 10 0

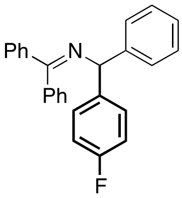
Ph
N
Ph
F

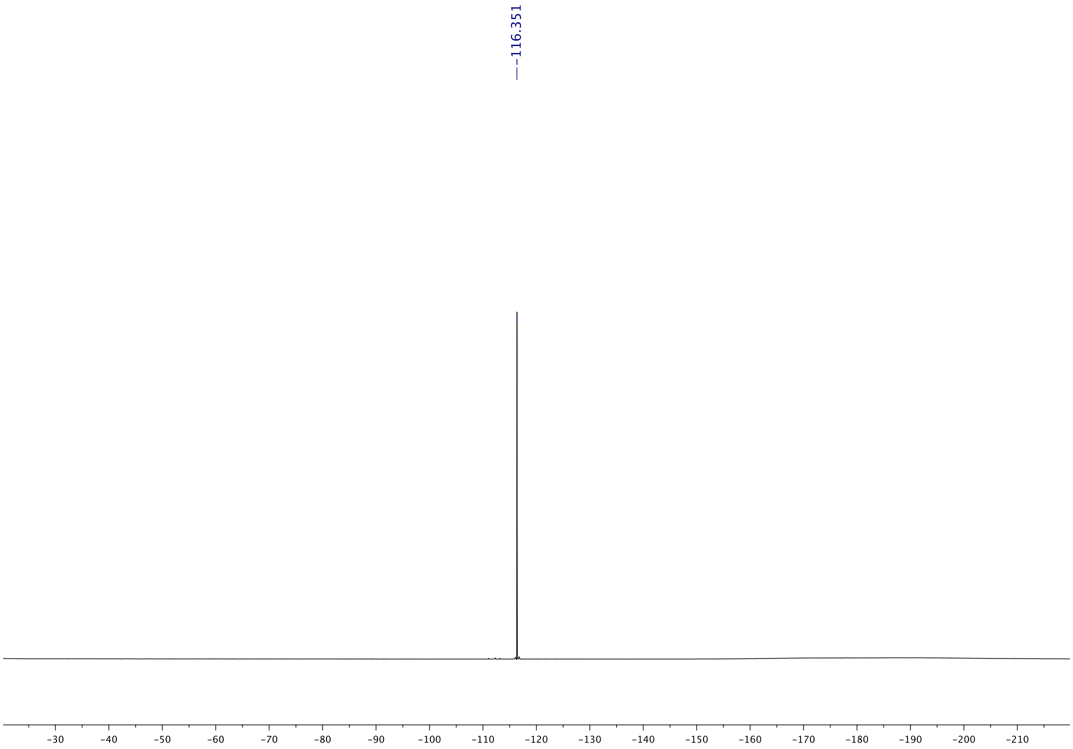
-116.351
-30
-40
-50
-60
-70
-80
-90
-100
-110
-120
-130
-140
-150
-160
-170
-180
-190
-200
-210

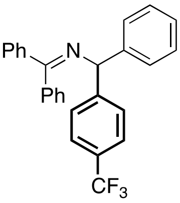

Ph
N
Ph
CF3

3d

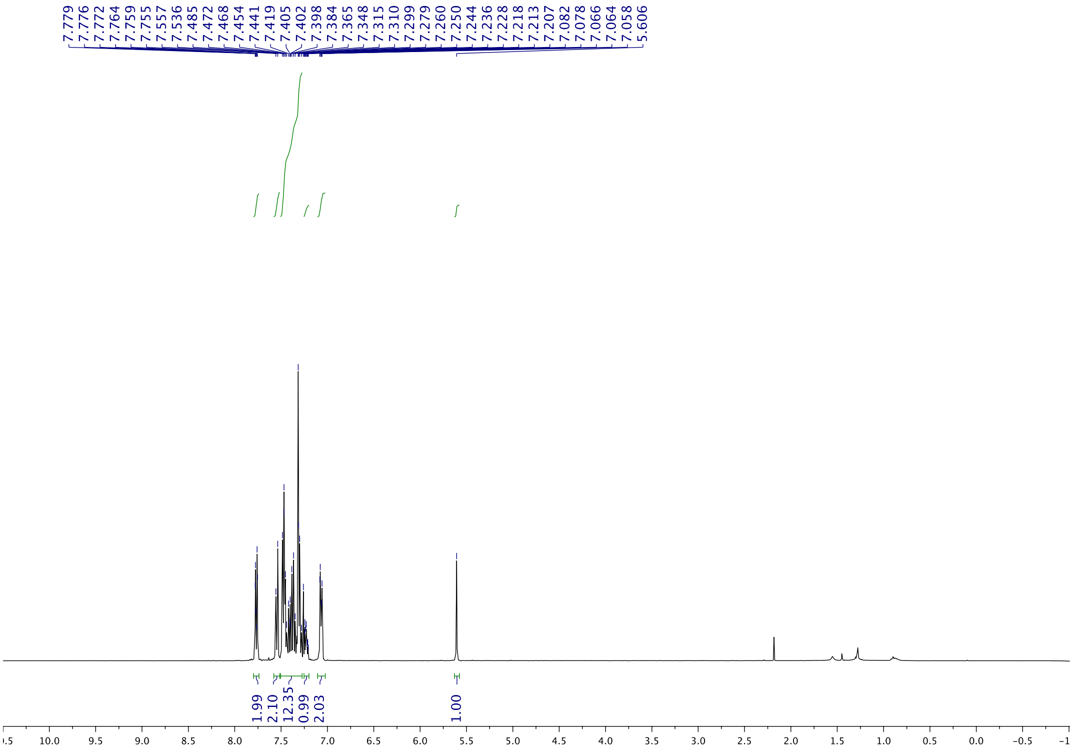

7.779
7.776
7.772
7.764
7.759
7.755
7.557
7.536
7.485
7.472
7.468
7.454
7.441
7.419
7.405
7.402
7.398
7.384
7.365
7.348
7.315
7.310
7.299
7.279
7.260
7.250
7.244
7.236
7.228
7.218
7.213
7.207
7.082
7.078
7.066
7.064
7.058
5.606
1.99
2.10
12.35
0.99
2.03
1.00
10.0 9.5 9.0 8.5 8.0 7.5 7.0 6.5 6.0 5.5 5.0 4.5 4.0 3.5 3.0 2.5 2.0 1.5 1.0 0.5 0.0 -0.5

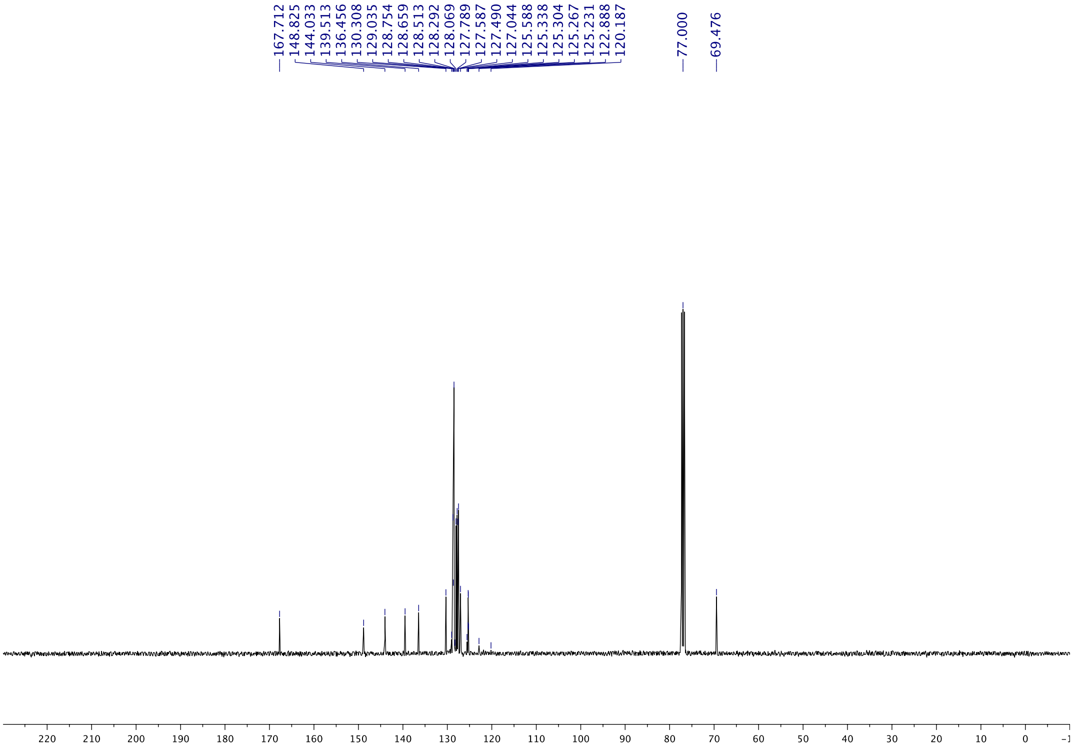

167.712
148.825
144.033
139.513
136.456
130.308
129.035
128.754
128.659
128.513
128.292
128.069
127.789
127.587
127.490
127.044
125.588
125.338
125.304
125.267
125.231
122.888
120.187
77.000
69.476
220 210 200 190 180 170 160 150 140 130 120 110 100 90 80 70 60 50 40 30 20 10 0

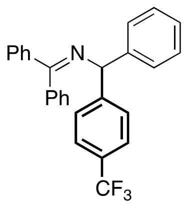
Ph
N
Ph
CF3

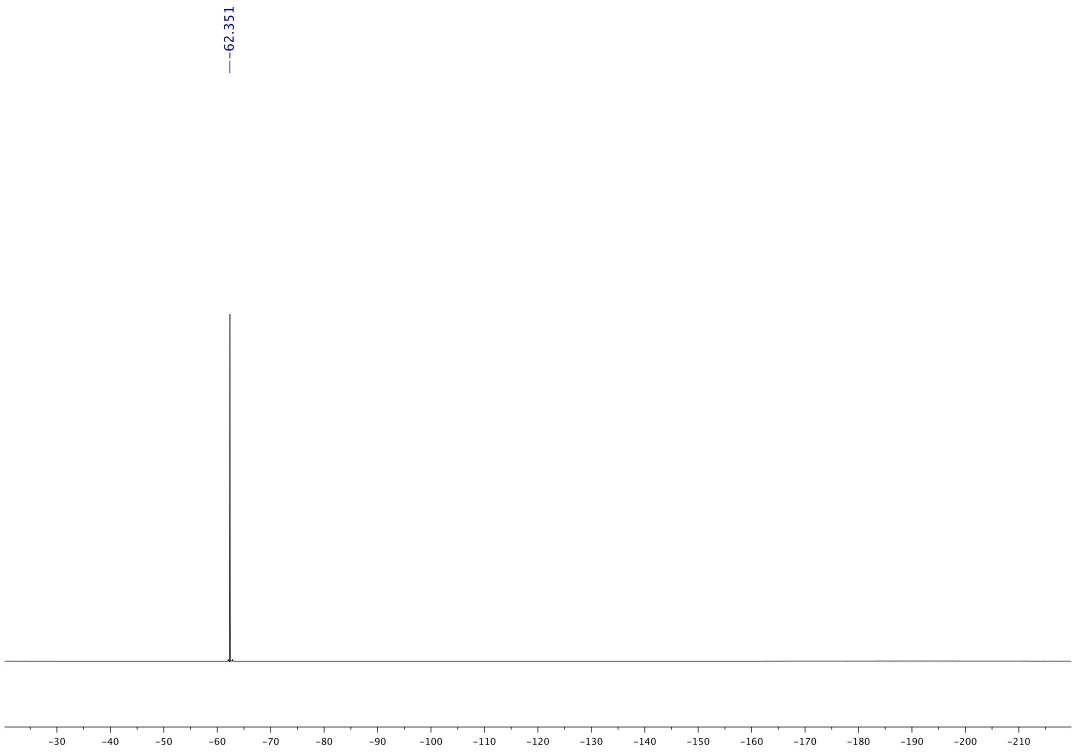
-62.351
-30
-40
-50
-60
-70
-80
-90
-100
-110
-120
-130
-140
-150
-160
-170
-180
-190
-200
-210

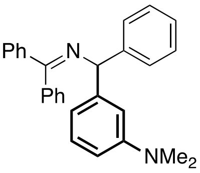
Ph
N
Ph
NMe2

3e

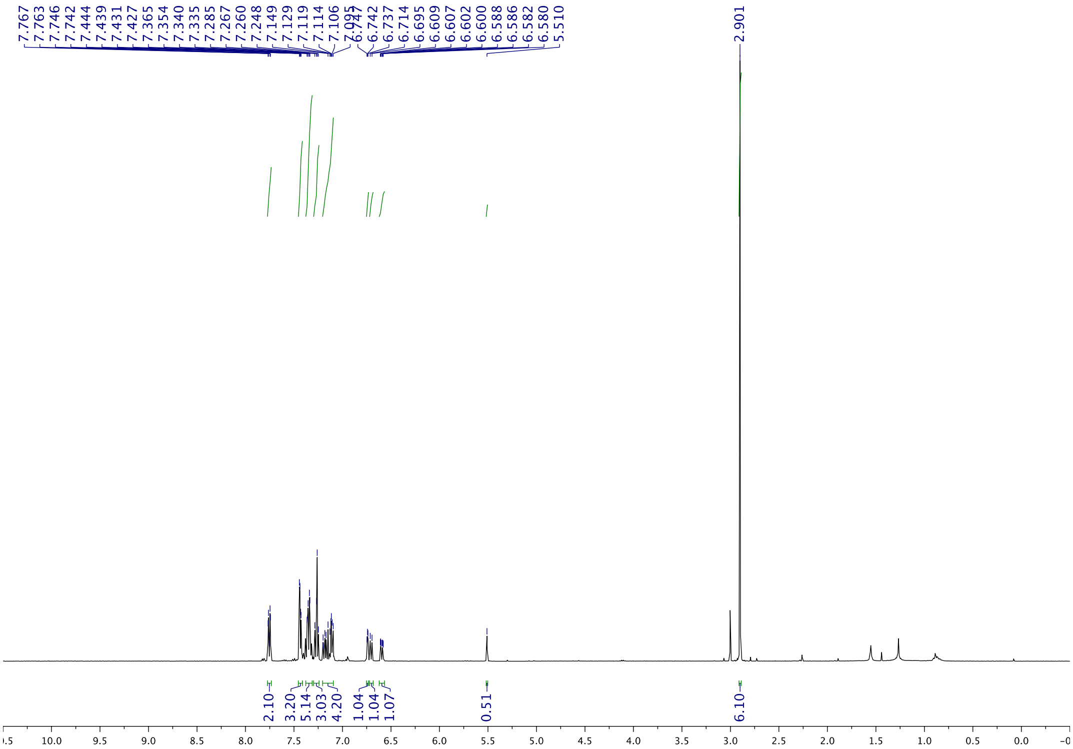
7.767
7.763
7.746
7.742
7.444
7.439
7.431
7.427
7.365
7.354
7.340
7.335
7.285
7.267
7.260
7.248
7.149
7.129
7.119
7.114
7.106
6.742
6.737
6.714
6.695
6.609
6.607
6.602
6.600
6.588
6.586
6.582
6.580
5.510
2.901
2.10
3.20
5.14
3.03
4.20
1.04
1.04
1.07
0.51
6.10

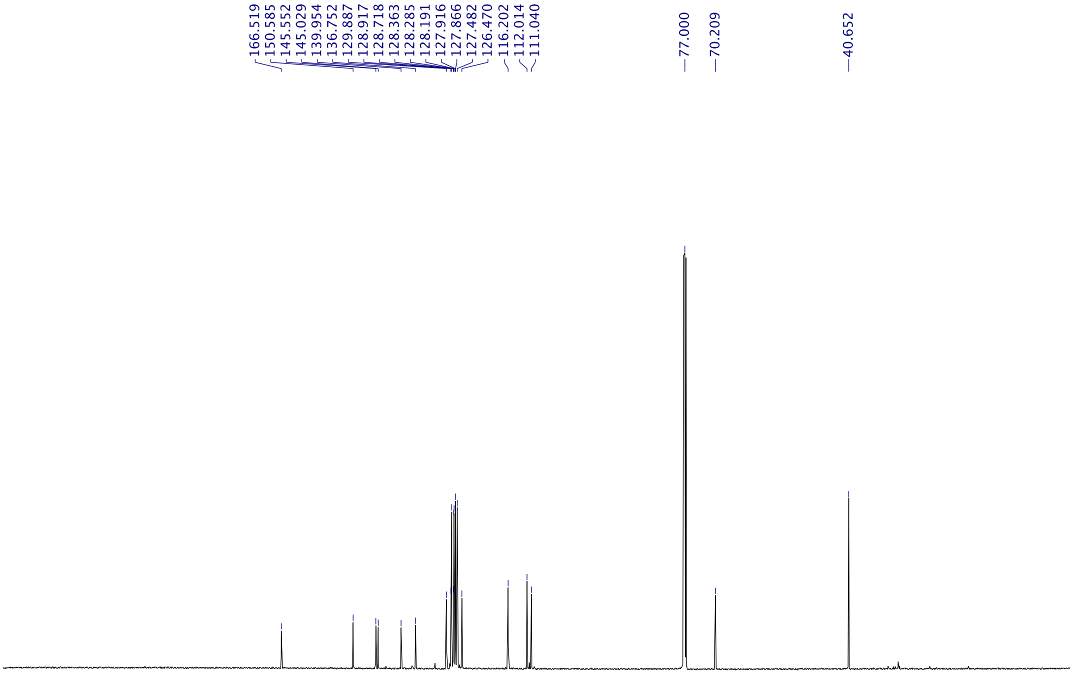
166.519
150.585
145.552
145.029
139.954
136.752
129.887
128.917
128.718
128.363
128.285
128.191
127.916
127.866
127.482
126.470
116.202
112.014
111.040
77.000
70.209
40.652

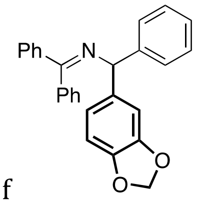
Ph
N
Ph
O
O

3f

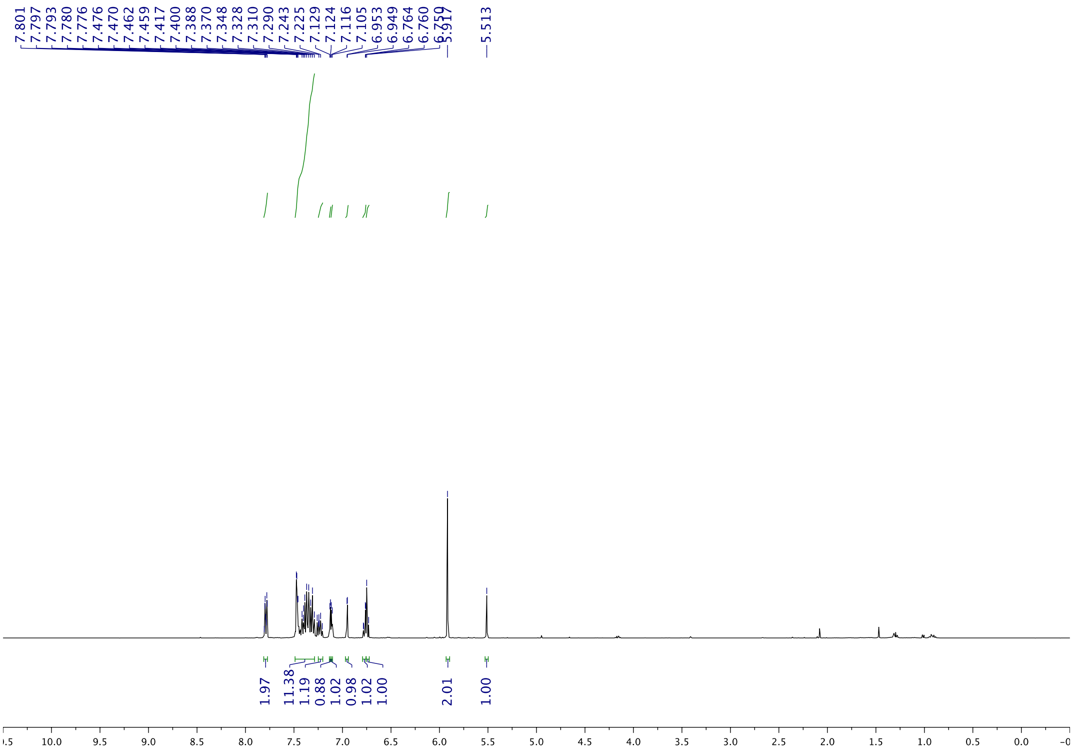
7.801
7.797
7.793
7.780
7.776
7.476
7.470
7.462
7.459
7.417
7.400
7.388
7.370
7.348
7.328
7.310
7.290
7.243
7.225
7.129
7.124
7.116
7.105
6.953
6.949
6.764
6.760
6.759
5.917
5.513
1.97
11.38
1.19
0.88
1.02
0.98
1.02
1.00
2.01
1.00
10.0 9.5 9.0 8.5 8.0 7.5 7.0 6.5 6.0 5.5 5.0 4.5 4.0 3.5 3.0 2.5 2.0 1.5 1.0 0.5 0.0

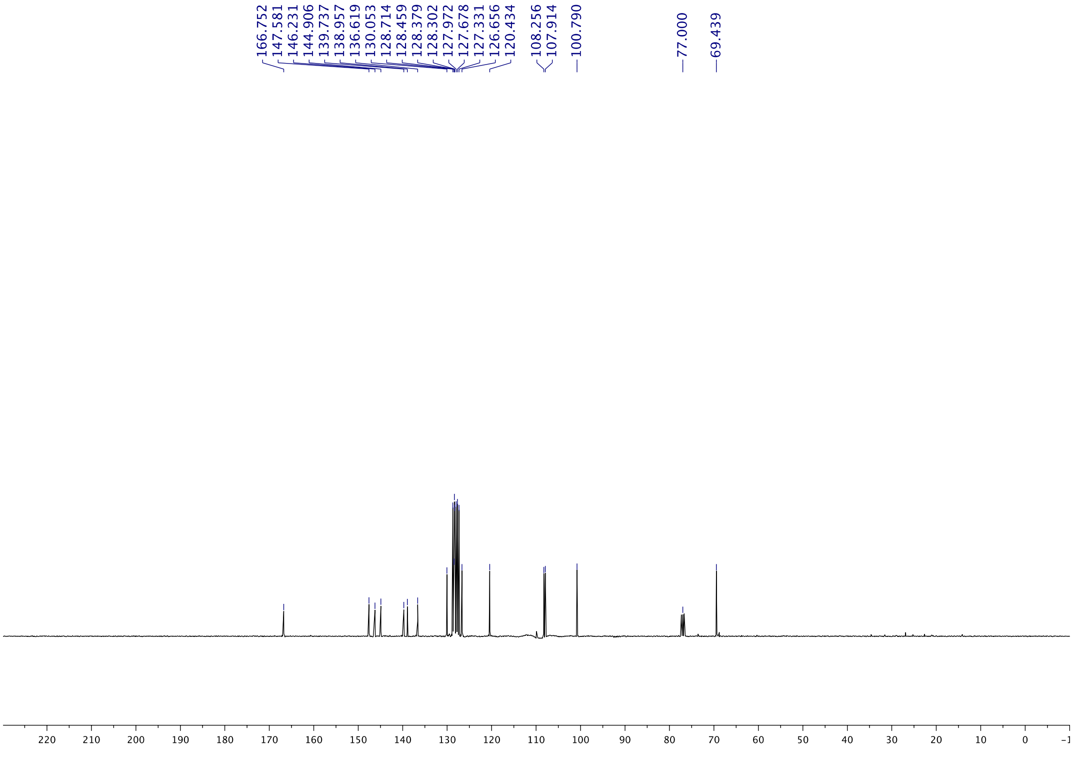
166.752
147.581
146.231
144.906
139.737
138.957
136.619
130.053
128.714
128.459
128.379
128.302
127.972
127.678
127.331
126.656
120.434
108.256
107.914
100.790
77.000
69.439
220 210 200 190 180 170 160 150 140 130 120 110 100 90 80 70 60 50 40 30 20 10 0

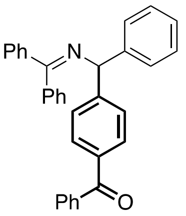
Ph
N
Ph
Ph
O

3g

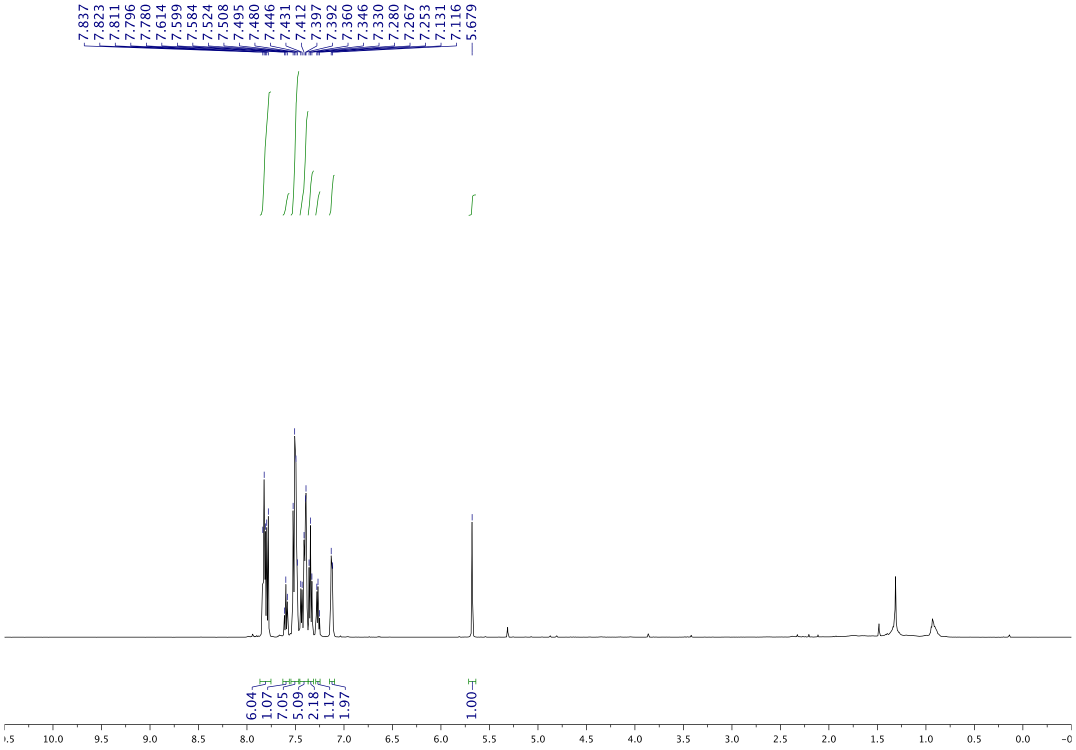
7.837
7.823
7.811
7.796
7.780
7.614
7.599
7.584
7.524
7.508
7.495
7.480
7.446
7.431
7.412
7.397
7.392
7.360
7.346
7.330
7.280
7.267
7.253
7.131
7.116
5.679
6.04
1.07
7.05
5.09
2.18
1.17
1.97
1.00
10.0
9.5
9.0
8.5
8.0
7.5
7.0
6.5
6.0
5.5
5.0
4.5
4.0
3.5
3.0
2.5
2.0
1.5
1.0
0.5
0.0

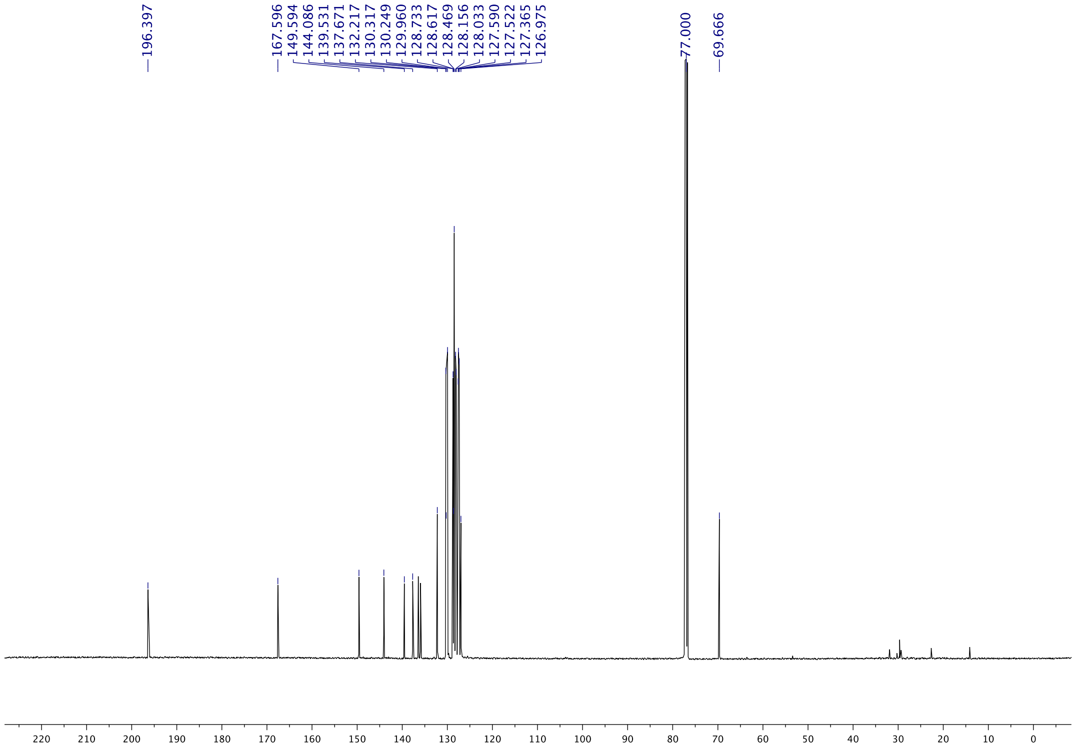
196.397
167.596
149.594
144.086
139.531
137.671
132.217
130.317
130.249
129.960
128.733
128.617
128.469
128.156
128.033
127.590
127.522
127.365
126.975
77.000
69.666
220
210
200
190
180
170
160
150
140
130
120
110
100
90
80
70
60
50
40
30
20
10
0

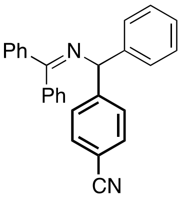
Ph
N
Ph
CN

3h

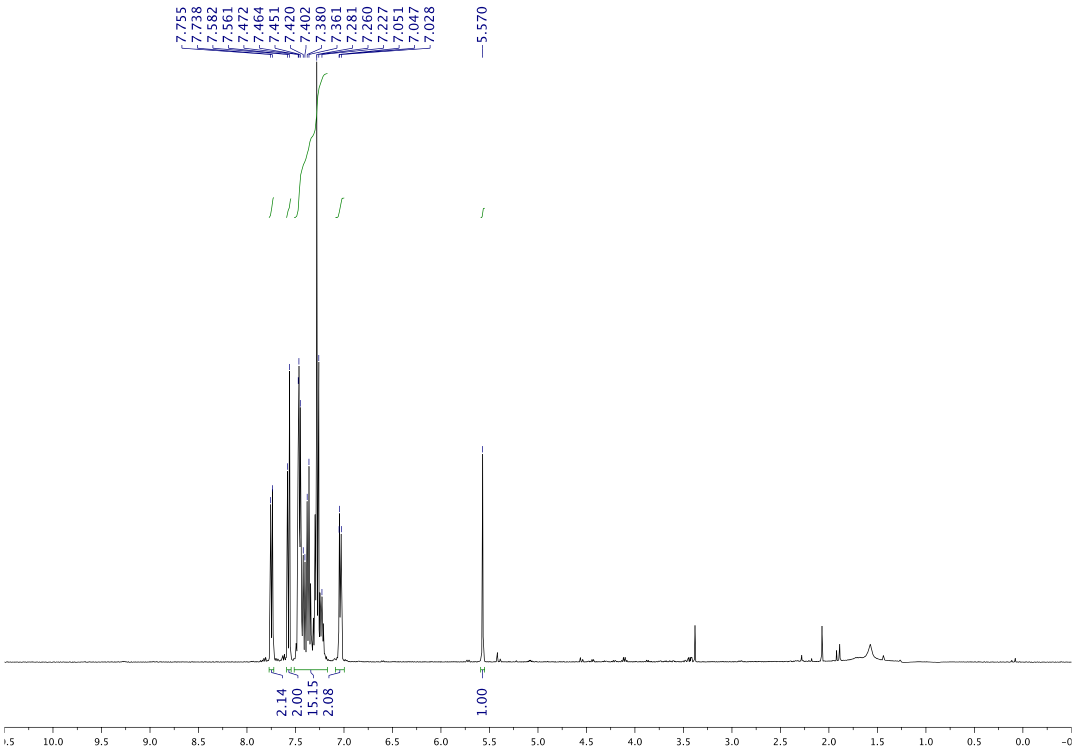
7.755
7.738
7.582
7.561
7.472
7.464
7.451
7.420
7.402
7.380
7.361
7.281
7.260
7.227
7.051
7.047
7.028
5.570
2.14
2.00
15.15
2.08
1.00

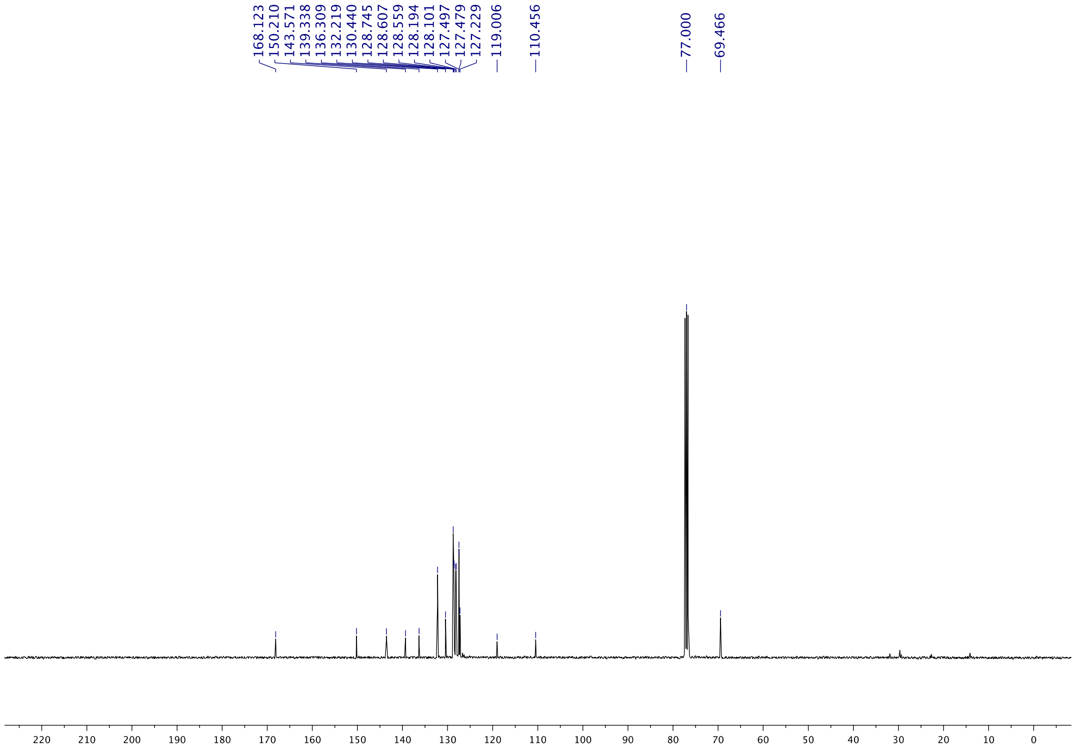
168.123
150.210
143.571
139.338
136.309
132.219
130.440
128.745
128.607
128.559
128.194
128.101
127.497
127.479
127.229
119.006
110.456
77.000
69.466

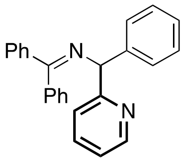
Ph
N
Ph
N

3i

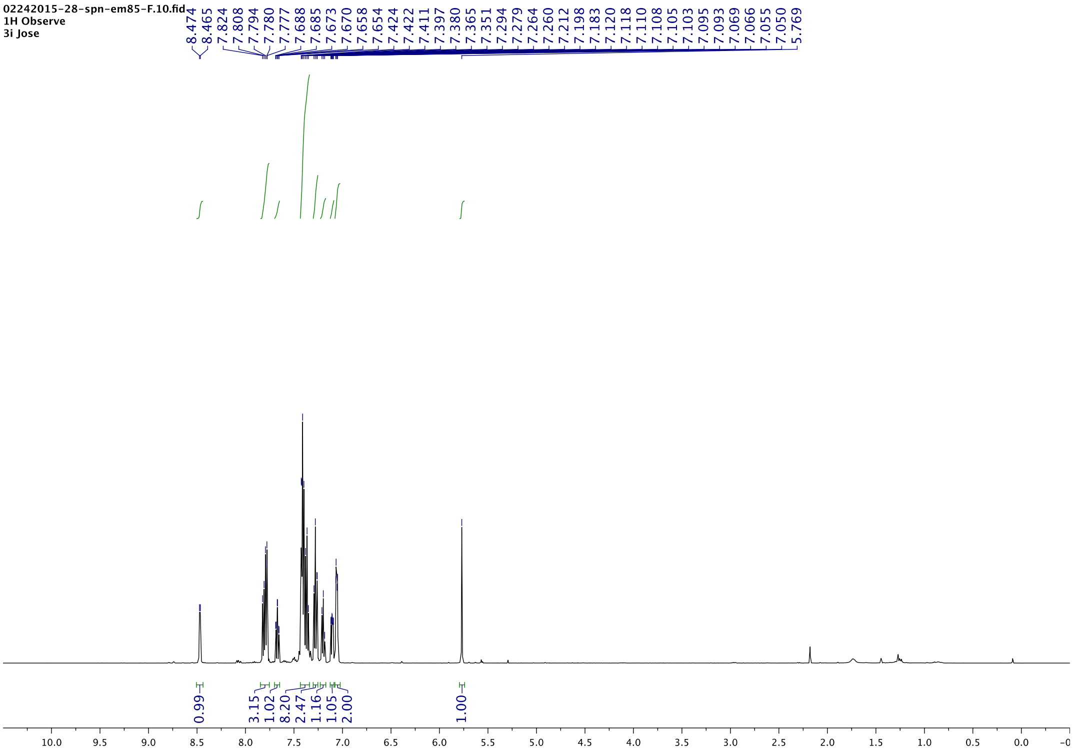
02242015-28-spn-em85-F.10.fid
1H Observe
3i Jose
8.474
8.465
7.824
7.808
7.794
7.780
7.777
7.688
7.685
7.673
7.670
7.658
7.654
7.424
7.422
7.411
7.397
7.380
7.365
7.351
7.294
7.279
7.264
7.260
7.212
7.198
7.183
7.120
7.118
7.110
7.108
7.105
7.103
7.095
7.093
7.069
7.066
7.055
7.050
5.769
0.99
3.15
1.02
8.20
2.47
1.16
1.05
2.00
1.00
10.0
9.5
9.0
8.5
8.0
7.5
7.0
6.5
6.0
5.5
5.0
4.5
4.0
3.5
3.0
2.5
2.0
1.5
1.0
0.5
0.0

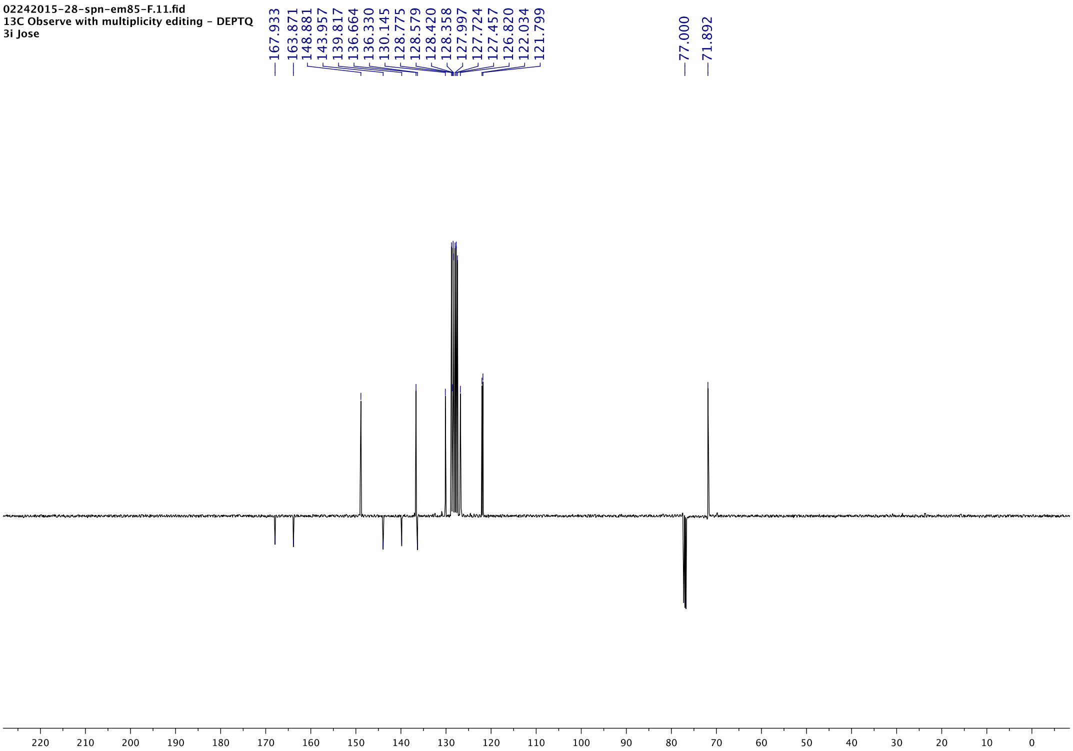
02242015-28-spn-em85-F.11.fid
13C Observe with multiplicity editing - DEPTQ
3i Jose
167.933
163.871
148.881
143.957
139.817
136.664
136.330
130.145
128.775
128.579
128.420
128.358
127.997
127.724
127.457
126.820
122.034
121.799
77.000
71.892
220
210
200
190
180
170
160
150
140
130
120
110
100
90
80
70
60
50
40
30
20
10
0

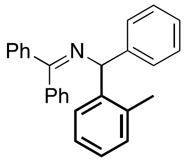
Ph
N
Ph

3j

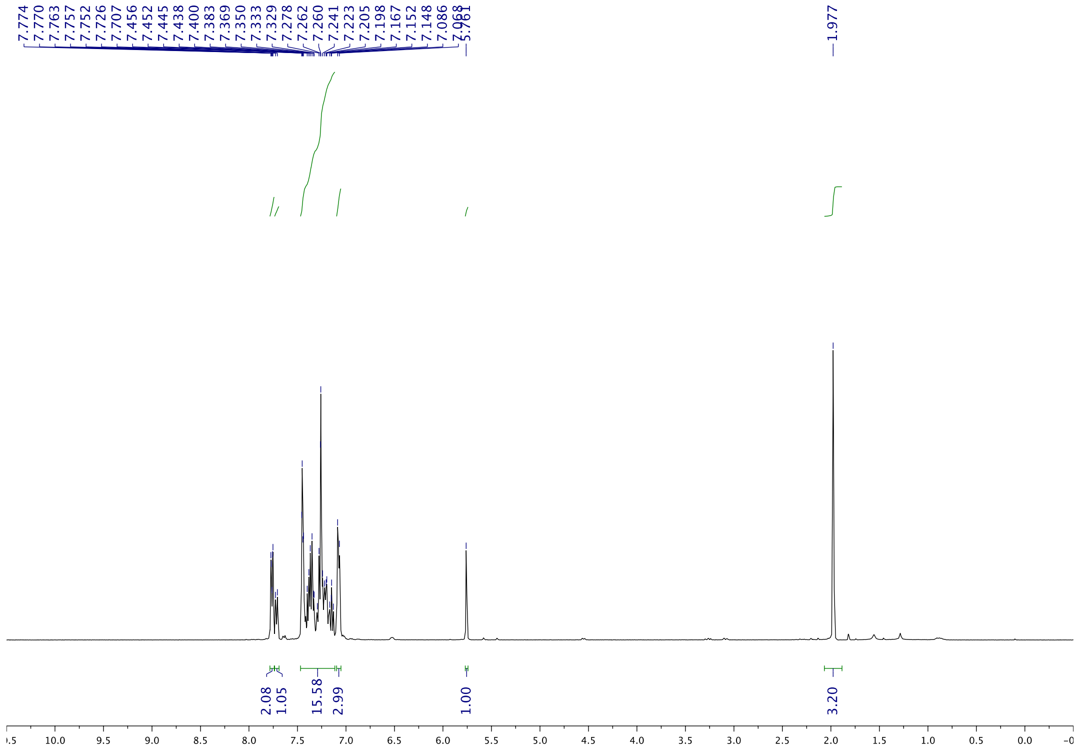
7.774
7.770
7.763
7.757
7.752
7.726
7.707
7.456
7.452
7.445
7.438
7.400
7.383
7.369
7.350
7.333
7.329
7.278
7.262
7.260
7.241
7.223
7.205
7.198
7.167
7.152
7.148
7.086
7.068
5.761
1.977
2.08
1.05
15.58
2.99
1.00
3.20
10.0 9.5 9.0 8.5 8.0 7.5 7.0 6.5 6.0 5.5 5.0 4.5 4.0 3.5 3.0 2.5 2.0 1.5 1.0 0.5 0.0

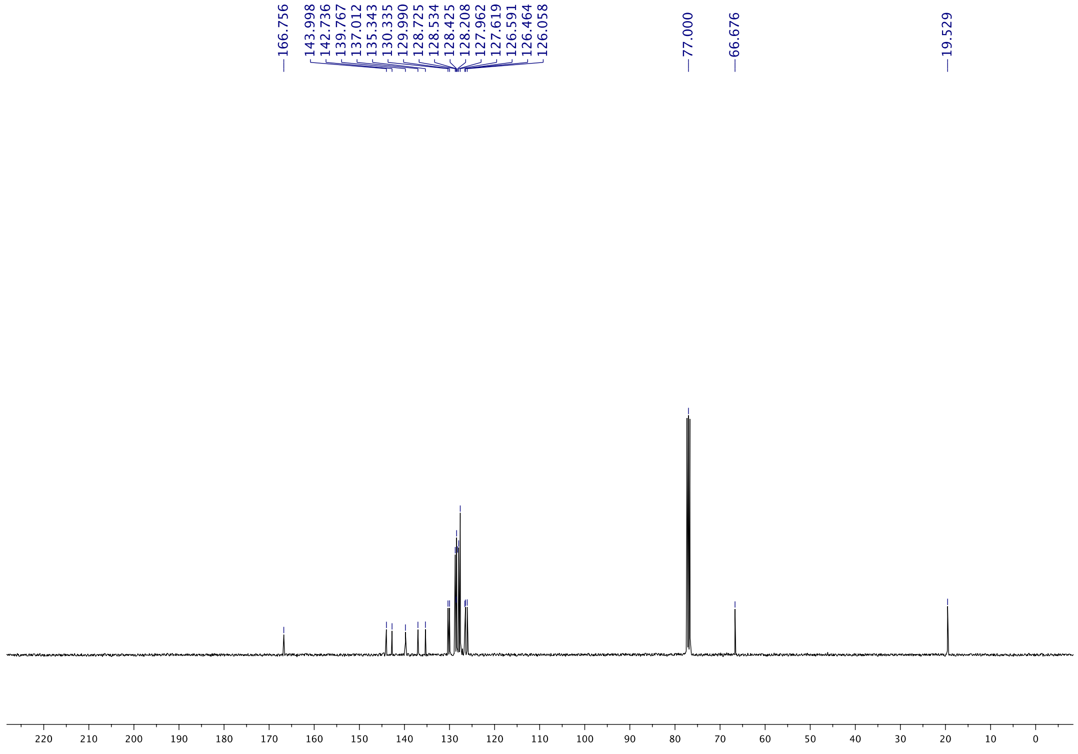
166.756
143.998
142.736
139.767
137.012
135.343
130.335
129.990
128.725
128.534
128.425
128.208
127.962
127.619
126.591
126.464
126.058
77.000
66.676
19.529
220 210 200 190 180 170 160 150 140 130 120 110 100 90 80 70 60 50 40 30 20 10 0

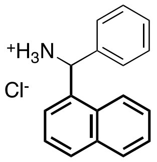

+H3N
Cl-

3k

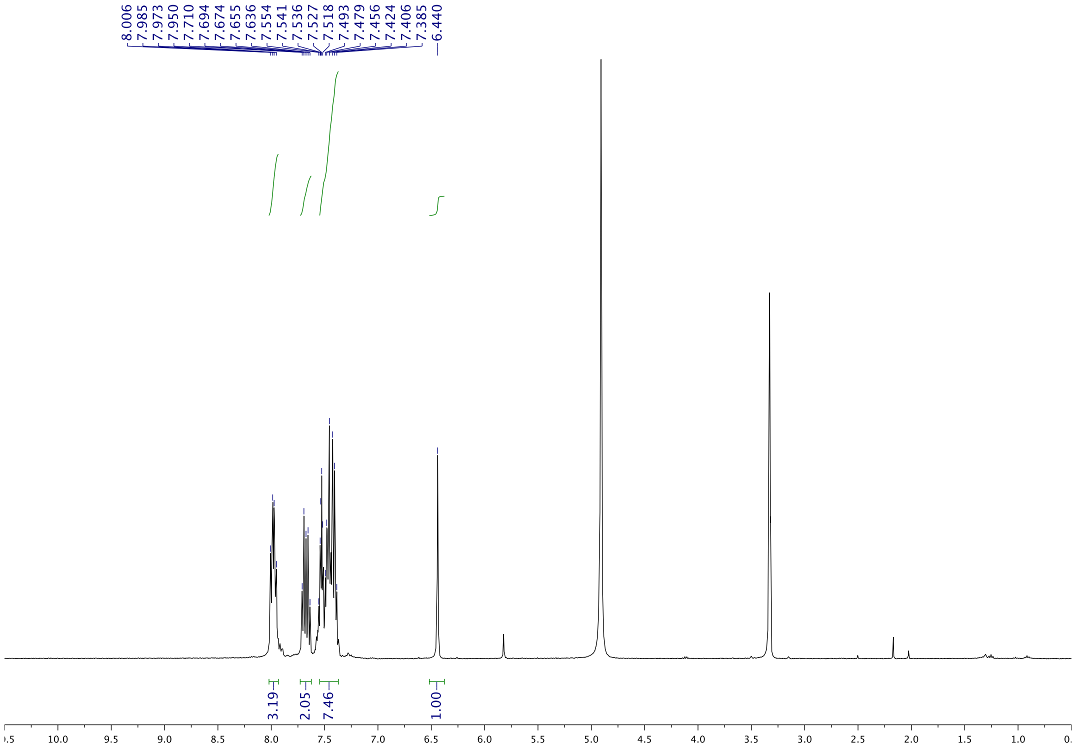

8.006
7.985
7.973
7.950
7.710
7.694
7.674
7.655
7.636
7.554
7.541
7.536
7.527
7.518
7.493
7.479
7.456
7.424
7.406
7.385
6.440
3.19
2.05
7.46
1.00
10.0
9.5
9.0
8.5
8.0
7.5
7.0
6.5
6.0
5.5
5.0
4.5
4.0
3.5
3.0
2.5
2.0
1.5
1.0
ppm

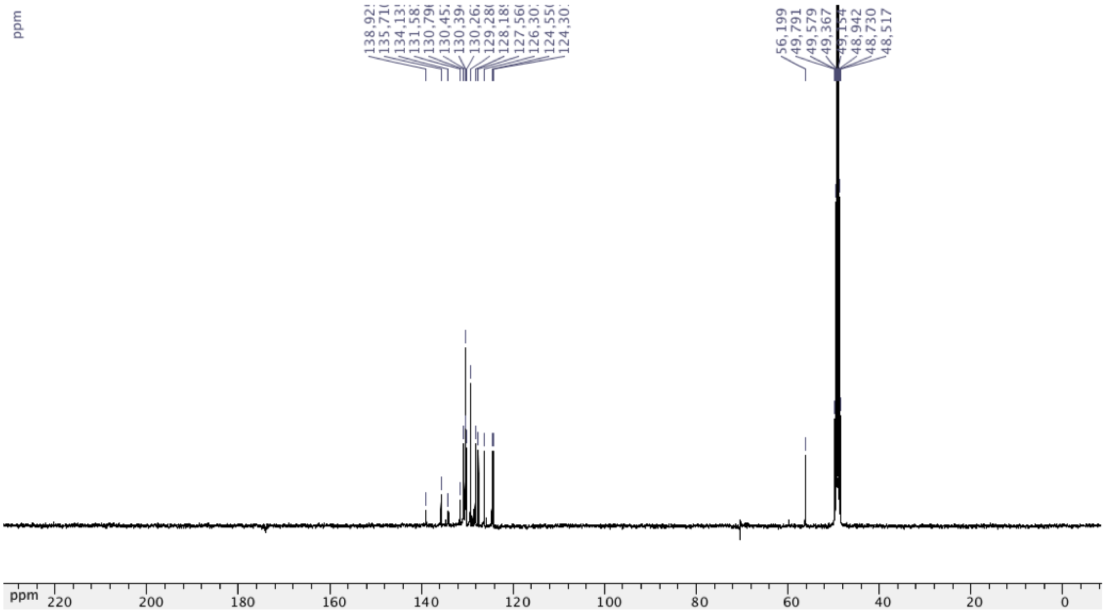

56,199
49,791
49,579
49,367
49,154
48,942
48,730
48,517
ppm
220
200
180
160
140
120
100
80
60
40
20
0
